# Mitigating aluminum toxicity and promoting plant resilience in acidic soil with *Penicillium olsonii* TLL1

**DOI:** 10.3389/fpls.2024.1423617

**Published:** 2024-06-20

**Authors:** Savitha Dhandapani, Yee Hwui Sng, Valiya Nadakkakath Agisha, Erinjery Jose Suraby, Bong Soo Park

**Affiliations:** Temasek Life Sciences Laboratory, 1 Research Link, National University of Singapore, Singapore, Singapore

**Keywords:** *Penicillium olsonii* TLL1 strain, aluminum toxicity, alleviate, acidic soil, internal detoxification, external exclusion

## Abstract

Aluminum (Al), prevalent in the crust of the Earth, jeopardizes plant health in acidic soils, hindering root growth and overall development. In this study, we first analysed the Al- and pH- tolerance of the *Penicillium olsonii* TLL1 strain (POT1; NRRL:68252) and investigated the potential for enhancing plant resilience under Al-rich acidic soil conditions. Our research illustrates the extraordinary tolerance of POT1 to both high Al concentrations and acidic conditions, showcasing its potential to alleviate Al-induced stress in plants. Metabolite analysis revealed that POT1 detoxifies Al through organic acid-dependent chelation mechanisms, significantly reducing Al stress in *Arabidopsis* and Pak Choi plants. Consequently, plant growth conditions improved, and the Al content in plant tissues decreased. Transcriptome analysis indicated that POT1 treatment downregulates genes associated with Al and oxidative stress such as *MATE, ALS3, NIP1–2* and several peroxidases, highlighting its effectiveness in lessening Al-induced damage. Comparative assessments highlight the superior performance of POT1 compared to other Al-tolerant Penicillium species, attributed to its ability to thrive in diverse pH levels and effectively detoxify Al. These findings position POT1 as a promising agent for enhancing crop resilience in Al-compromised acidic soils, offering new avenues for promoting plant health and bolstering food security through increased crop yield and safety.

## 1 Introduction

Aluminum (Al) is abundant in the crust of the Earth and poses a significant threat to plant health, particularly in acidic soils with a pH below 5.5 (Kochian, 1995; Kochian et al., 2015). With approximately half of the world’s arable lands being acidic (von Uexküll and Mutert, 1995; Kochian et al., 2015), various factors such as the use of acid-forming fertilizers, air pollution, and industrialization have further compounded this issue. These anthropogenic actions have also played a role in worsening climate change, which in turn will reduce plant growth and agricultural productivity due to increasingly frequent occurrences of water scarcity. This impact will be especially notable in tropical regions, where soil acidity and heightened Al concentrations are inherent features resulting from soil weathering processes (Cruz, 2023). Consequently, it is essential to adopt soil management techniques aimed at mitigating the detrimental effects of Al stress on plants.

Al levels are commonly observed to be relatively elevated in dried grains and leafy vegetables (Liang et al., 2019). Leafy vegetables such as spinach, lettuce, and Pak Choi are globally consumed and vital for human nutrition, providing vitamins A, C, folic acid, minerals, and fiber with low fat and carbohydrate content. However, these vegetables, including spinach and Pak Choi, are known as significant Al accumulators from soil and water, posing potential health risks (Verstraeten et al., 2008; Kandimalla et al., 2016). In humans, high intake of Al has been associated with neurotoxicity, bone disorders, and potential neurodegenerative diseases such as Alzheimer’s (Exley, 2013). Therefore, it is crucial to monitor Al levels in food crops and implement measures to mitigate exposure to excessive Al intake. Traditional solutions involve lime and magnesium application to raise pH and mitigate toxicity, but drawbacks include zinc and manganese deficiencies, along with magnesium toxicity (Guo et al., 2006; Bose et al., 2011; Jayaganesh et al., 2011). Moreover, economic constraints in many target countries may further hinder the implementation of these strategies (von Uexküll and Mutert, 1995).

Excessive Al levels diminish plant height, fresh and dry weights. Lateral roots become stunted, and the entire root system fails to elongate (Kopittke and Blamey, 2016). Previous studies have shown decreased root growth rates, shorter roots, and reduced masses in plants exposed to Al within a pH range of 4.2 to 5.4 (Kochian et al., 2015; Sade et al., 2016). Under high Al conditions, the root apex is the primary site of injury, exhibiting symptoms like irregular cell division (Jaskowiak et al., 2018; Yan et al., 2018), cell wall thickening, callose deposition, and reactive oxygen species (ROS) production (Huang et al., 2014; Wu et al., 2022). Plant species exhibit varying responses to Al stress due to differences in tolerance or sensitivity (Ezaki et al., 2000). While certain crops like pineapple and tea exhibit tolerance to elevated levels of Al, it remains a significant constraint for most of the crops. Plants employ two primary mechanisms to resist Al toxicity: preventing Al from entering the root apex and detoxifying Al by sequestering it in vacuoles. This involves the release of organic acids (OAs) such as malate, citrate, and oxalate into the soil, which chelate Al^3+^ and render them non-phytotoxic (Brunner and Sperisen, 2013; Kichigina et al., 2017; Yan et al., 2022b). Transporter proteins on the plasma membrane and vacuole ABC transporters play crucial roles in controlling the uptake and sequestration of Al^3+^ into vacuoles, enhancing plant resistance to Al (Huang et al., 2009; Liu et al., 2009; Xia et al., 2010).

The application of exogenous regulatory factors, such as boron, organic acids, amino acids, phytohormones, and biochar, has shown considerable potential in alleviating Al toxicity in plants by modulating various physiological and biochemical pathways and enhancing antioxidant defense mechanisms (Yan et al., 2021, 2022a, 2023). These factors promote the secretion of organic acids and reduce Al deposition in cell walls, thereby enhancing plant resilience in acidic soils. As a result, the use of these external factors not only improves crop yield and quality but also supports sustainable agricultural practices and food security. In line with this approach, research into plant growth-promoting fungi (PGPF) has gained significant attention due to their ability to enhance plant growth and mitigate the harmful effects of heavy metal toxicity and adverse soil conditions (Ma et al., 2015; Khan et al., 2017). Elevated Al concentrations impede fungal growth (He et al., 2016), while low pH inhibits spore germination and hyphal growth of many PGPF (Porter et al., 1987). Despite these obstacles, certain *Penicillium* species exhibit Al- and pH- resistance (Kawai et al., 2000; Zhang et al., 2002; Dhakar et al., 2014; He et al., 2016). Some fungi have shown tolerance to high concentrations of Al and employ various resistance mechanisms, such as symplastic tolerance and Al exclusion using low molecular weight Al-chelating ligands (Kochian, 1995; Kawai et al., 2000). For instance, certain *Penicillium* strains can exude citric acid in Al-rich environments, effectively alleviating Al toxicity in plants (Zhang et al., 2002). PGPF have shown promising results in alleviating heavy metal stress and promoting plant growth through mechanisms such as phytohormone production and stress tolerance induction (Hassan et al., 2014). Thus, identifying new PGPF and understanding their interactions with plants under Al-rich, acidic soil conditions promise further advancements in sustainable agriculture and environmental restoration.

Previous research has confirmed the phosphorus-solubilizing activity of *Penicillium olsonii* TLL1 (POT1), promoting rice growth in vermiculite and in insoluble phosphate abundant soil indigenous to Singapore (Suraby et al., 2023). In this study, we evaluated the Al and pH tolerance of the POT1 strain and investigated its role in enhancing plant growth resilience under high-Al and acidic soil conditions. Our research sheds light on some of the mechanisms underlying the plant growth promoting effects of POT1 and its potential applications in agriculture and ecosystem restoration under climate change. Additionally, we compared its efficacy against other metal ion-tolerant and plant growth-promoting *Penicillium* strains.

## 2 Materials and methods

### 2.1 Al tolerance assay

Stock solution of aluminum chloride was filter sterilized and mixed with autoclaved malt extract agar (MEA) to obtain final concentrations of 0.2, 1, 2, 5, 10, and 20mM. Control plates without AlCl_3_ were also prepared using MEA. 25mL mixture was poured into 9cm diameter petri dishes. POT1 spores were inoculated onto the media, and diameters were measured after 7 days of incubation at room temperature.

### 2.2 pH tolerance assay

Malt extract broth (MEB) ranging from pH 1 to 11 was autoclaved, inoculated with POT1, and cultured for 14 days. The mycelia from each flask were subsequently harvested and oven-dried at 80°C to measure dry weight.

### 2.3 Plant materials and germination

Surface-sterilized seeds of wild-type *Arabidopsis thaliana* (Col-0) and Pak Choi (*Brassica rapa* var. *chinensis*) were sown on agar medium containing Murashige and Skoog (MS) basal salts, 1% (w/v) sucrose, and 2.5mM MES at pH 5.6. After 2 days of imbibition at 4°C in the dark, seedlings were germinated in a tissue culture chamber under conditions of 22°C, 60% relative humidity, and a 16h light/8h dark cycle with a light intensity of 100µmol·m^−2^·s^−1^.

### 2.4 Effect of POT1 on *Arabidopsis* growth in Al-containing media

Four-day-old *Arabidopsis* seedlings were transferred to pH 4.0 and pH 5.6 modified Hoagland’s nutrient medium with 1.5% (w/v) phyto agar (Millner and Kitt, 1992) for the following treatments: (1) Control, (2) Al (100µM AlCl_3_), and (3) Al+POT1 (POT1 inoculated on Hoagland’s medium containing 100µM AlCl_3_ and grown for 1 week before transferring the seedlings). The modified Hoagland’s nutrient comprised 2.5mM Ca(NO_3_)_2_, 2.5mM KNO_3_, 1mM MgSO_4_, 0.75µM NaFeEDTA, 0.25mM KH_2_PO_4_, 50µM H_3_BO_3_, 0.075 µM NH_4_Mo_7_O_24_, 2µM ZnSO_4_, 10µM MnCl_2_, 1.5µM CuSO_4_, 0.2µM CoCl_2_, and 0.5mM MES, with all chemicals obtained from Sigma-Aldrich. The seedlings were grown vertically for 10 days in a tissue culture chamber under previously described conditions. Comparative studies were conducted using four other *Penicillium* species: *P. bilaiae* (ATCC 20851), *P. chrysogenum* (NBRC 4626), *P. janthinellum* (NBRC 31133), and *P. simplicissimum* (NBRC 106922).

### 2.5 Effect of POT1 on plant growth in Al-containing soil

Two-week-old *Arabidopsis* and one-week-old Pak Choi seedlings were transplanted from germinating medium to soil. For POT1 induction treatments, each soil pot received 0.5g of freshly grown and rinsed POT1 mycelia one week before seedling transplant. Plants were grown for four weeks in a growth room under conditions of 23°C, 60% relative humidity, and a 16h light/8h dark cycle with a light intensity of 100µmol·m^−2^·s^−1^.

Water containing different concentrations (0–100mM) of AlCl_3_ was used for Al treatment of *Arabidopsis* and Pak Choi plants. To simulate the natural occurrence of AlCl_3_ in acidic soils, the pH levels of AlCl_3_ solutions at various concentrations were not adjusted. This approach aimed to replicate the *in vivo* conditions of high Al content in acidic soils and to mimic the natural leachate of Al-contaminated soils at corresponding concentrations and pH levels. The pH values of the AlCl_3_ solutions were recorded as follows: 100mM-3.26; 50mM-3.55; 40mM-3.63; 30mM-3.73; 20mM-3.95; 10mM-4.02. *Arabidopsis* were watered twice per week, while Pak Choi plants were watered thrice per week. Control plants were watered with plain tap water.

### 2.6 Determination of fungal colonization by microscopy

*Arabidopsis* seedlings were grown with POT1 for 10 days. Roots were fixed in a 3:1 solution of ethanol to glacial acetic acid for 2h at 10 days post-inoculation. Fixed root tips were then incubated at 85°C for 10 minutes in 10% (w/v) KOH, followed by four washes with PBS (pH 7.4). To visualize fungal colonization in roots, root tips were double-stained with 10µg/mL Wheat Germ Agglutinin-Alexa Fluor 488 conjugate (WGA-AF488; 488_Ex_/500–540_Em_ nm; Invitrogen™, Waltham, Massachusetts, USA) for fungal structures and 20µg/mL propidium iodide (PI; 561_Ex_/580–630_Em_ nm; Sigma-Aldrich, St. Louis, Missouri, USA) for plant cell walls. Staining was done by vacuum infiltration following the previously described procedure (Redkar et al., 2018). Finally, samples were analyzed with a Leica SP8 confocal microscope (Leica; Wetzlar, Germany) equipped with an appropriate set of excitation/emission filters.

### 2.7 Measurement of chlorophyll and anthocyanin content

100mg of leaves were extracted overnight at 4°C using 10mL of 80% (v/v) acetone and the total chlorophyll levels were assessed following the previously described method (Porra et al., 1989). 100mg of leaves were extracted overnight at 4°C using 10mL of methanol-HCl (99:1) and the total anthocyanin levels were determined following the previously described procedure (Rabino and Mancinelli, 1986).

### 2.8 Measurement of elemental content

To analyze carbon (C), hydrogen (H), nitrogen (N), and sulfur (S) contents, *Arabidopsis* shoots and roots were dried at 60°C, ground into a fine powder, and assessed using an organic elemental analyzer (CHNS-O vario EL cube, Heraeus Elementar, Hanau, Germany). Flash combustion of 50mg of dried samples was performed for rapid oxidation, and the resulting combustion products were separated using a chromatographic column and detected with a thermal conductivity detector.

For analysis of remaining elements, 50mg of dried plant samples and POT1 pellets were digested with the addition of 5mL of HNO_3_ (68%) and 1mL of HCl (37%), followed by incubation at 220°C for 2h using graphite digestion equipment (DigestLinc ST60D). For analysis of growth media, 5mL of filtered media was digested as described above. The digested samples were naturally cooled to room temperature, diluted to 10mL with deionized water, and analyzed using Agilent 720 Inductively Coupled Plasma – Optical Emission Spectrometry (ICP-OES; Agilent, Santa Clara, California, USA) with a detection limit of 0.02ppm.

### 2.9 Al and callose staining

Al staining was performed by following the previously described method (Zheng et al., 2005). *Arabidopsis* seedling root tips from Control, Al, and Al+POT1 conditions were excised, washed in deionized water for 10min, and stained with 100μM morin (420_Ex_/515_Em_ nm, Sigma-Aldrich) in 10mM MES buffer, pH 5.5 for 30min. Stained root tips were then washed twice with MES buffer and deionized water for 5min each, mounted in 15% glycerol, and observed under ZEISS AXIOPLAN 2 (Carl Zeiss, Oberkochen, Baden-Württemberg, Germany) with appropriate excitation/emission filters.

Callose staining followed the previously described method (Clay et al., 2009) with modifications. Root tips were washed in 150mM K_2_HPO_4_ for 10min, stained with 150mM K_2_HPO_4_ and 0.01% (w/v) aniline blue (370_Ex_/509_Em_ nm; Sigma-Aldrich) for 1h in darkness, and callose depositions were visualized using an FV3000 confocal laser scanning microscope (Olympus) with suitable excitation/emission filters.

### 2.10 RNA sequencing and analysis

Total RNA from Al-treated and Al+POT1-treated *Arabidopsis* roots and leaves (three biological replicates each) was extracted using the FavorPrepTM Plant Total RNA Purification Kit (Favorgen, Taiwan). The RNA samples underwent quality checks with Nanodrop, agarose gel electrophoresis, and Agilent 2100 Bioanalyzer by Novogene (Singapore). RNA sequencing, performed on Illumina Novaseq 6000 with 150bp paired-end sequencing and an average sequence depth of 87M reads per sample (12 Gb/sample), mapped the reads to the *Arabidopsis thaliana* reference genome, ensuring >7.6M reads mapped for all samples. Differential expression analysis for Al-treat and Al+POT1-treated roots and shoots employed DESeq2, and statistical Gene Ontology (GO) enrichment analysis was conducted using the R package clusterProfiler, considering GO terms with adjusted *p*-values less than 0.05 as significantly enriched. The protein-protein interaction (PPI) networks for upregulated and downregulated DEGs in the roots and shoots were generated using the STRING database (http://www.string-db.org), employing gene IDs as input and a confidence level threshold of 0.4 (medium confidence).

### 2.11 Quantitative real-time PCR

cDNA synthesis and qRT-PCR were carried out as described in existing research (Dhandapani et al., 2023). The primers used in this study are given in **Supplementary Table 1**.

### 2.12 Organic acid analysis

POT1 spores were grown in MEB for 4 days, filtered, and 5g of cell pellets were introduced into MEB containing 100µM AlCl_3_ for 14 days. The negative control was POT1 grown in plain MEB. Spent media (5mL) was mixed with 5mL of chloroform and 12.5mL of methanol. Extraction and derivatization of organic acids (OAs) in the spent media followed the previously described method (Jeon et al., 2018). GC-TOF-MS analysis was conducted as previously described (Dhandapani et al., 2020) with the following modifications. The CP-Sil8 column was initially held at 80°C for 2min, ramped to 320°C at 15°C/min, and finally held at 320°C for 10min. MS measurements were performed in scan mode with a scan range of m/z 50 to 600.

### 2.13 Statistical analysis

Graphs, calculations, and statistical analyses were conducted using GraphPad Prism software version 9.5 for Windows (GraphPad Software, San Diego, CA, USA). Student’s *t*-test in Microsoft Excel was employed to determine the statistical significance of differences between the control and treatments, with *p* values less than 0.05 considered statistically significant. Figures present mean ± standard deviations.

## 3 Results

### 3.1 Al- and pH- tolerant POT1 mitigated Al stress and altered nutrient uptake in *Arabidopsis*

**Figure 1:**
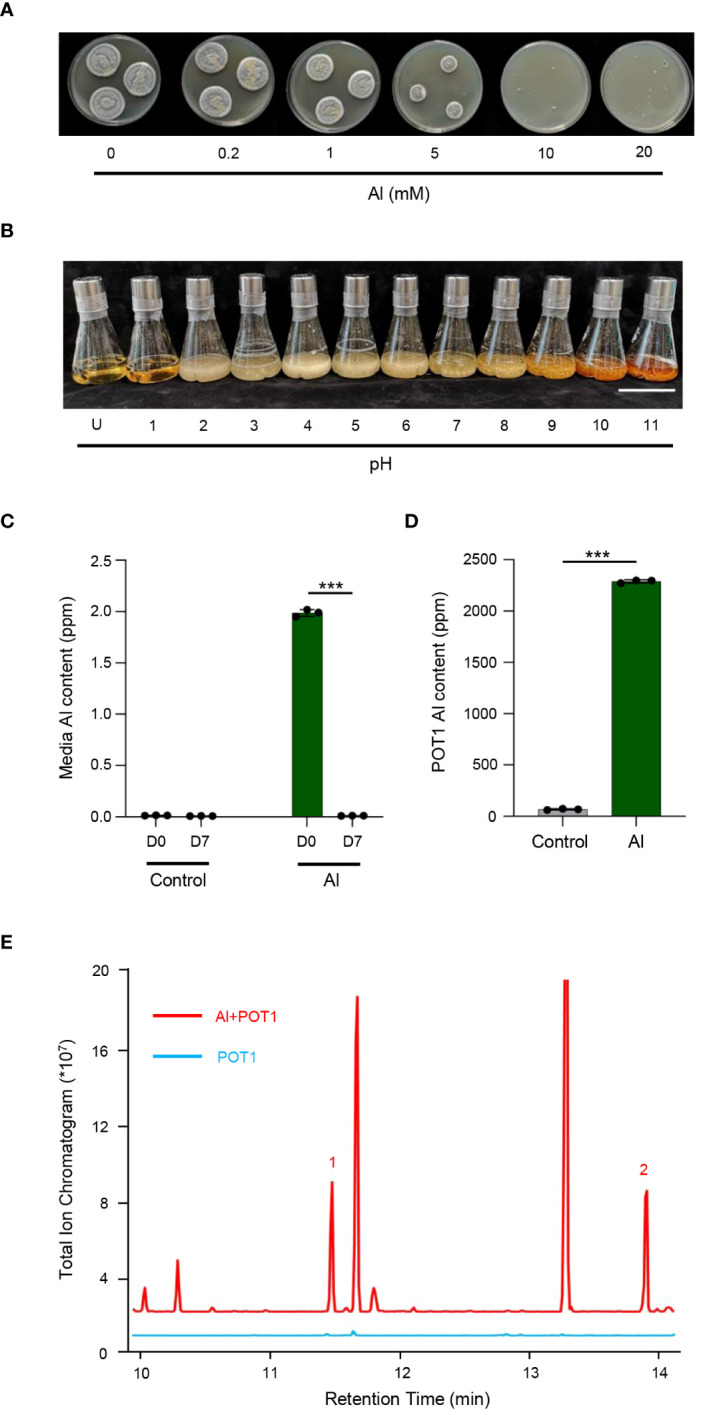
POT1 has Al and pH tolerance, and it detoxifies Al^3+^ by internal absorption and external sequestration. **(A)** Representative images of POT1 on malt extract agar supplemented with varying concentrations of AlCl_3_ (0.2–20 mM) compared to the control (0 mM). **(B)** Representative images of POT1 in malt extract broth at pH levels ranging from 2 to 11, illustrating its pH tolerance. “U” denotes uninoculated plain medium. Scale bar: 10 cm. **(C)** Comparison of Al content in spent media between control (0 µM AlCl_3_) and Al-treated (100 µM AlCl_3_) media on day 0 (D0) and day 7 (D7). **(D)** Assessment of Al content in POT1 mycelia cultured in control or Al-treated media on D7. Columns represent mean ± SD of three independent experiments. Statistical significances were determined using Student’s *t*-test (*** *p* < 0.001). Detailed *p* values are provided in **Supplementary Table 2**. **(E)** Total ion chromatogram of POT1 spent media on D7, indicating the production of malic acid (1) and citric acid (2) in the presence of Al (red; Al + POT1) but not in its absence (blue; POT1).

Testing for Al and pH tolerance revealed resilience of POT1 to high concentrations of Al (up to 5mM) and a broad pH range (pH 2 to 11) (**Figures 1A, B**; **Supplementary Figure 1**). Assessment of Al internalization revealed decreased Al content in the spent medium after 7 days of POT1 cultivation in Al-rich media, contrasting with mycelia grown under the same conditions which exhibited notably higher Al content (**Figures 1C, D**). Furthermore, malic acid and citric acid were detected in spent media of POT1 grown with Al, suggesting a potential role of OAs in detoxifying Al^3+^ (**Figure 1E**).

**Figure 2:**
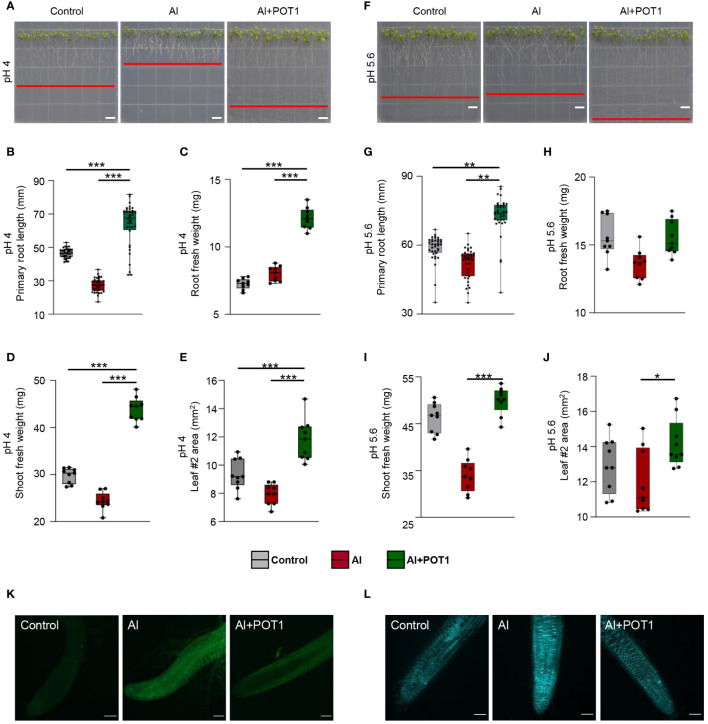
POT1 alleviates Al stress in acidic conditions. Visual representations of *Arabidopsis* seedlings subjected to different treatments: control (0µM AlCl_3_), Al stress (100µM AlCl_3_), or Al stress with POT1 colonies (Al+POT1) at pH 4 **(A)** and pH 5.6 **(F)**. Red lines indicate the longest root position. Scale bar: 1 cm. Evaluation of primary root growth **(B, G)**, root fresh weight **(C, H)**, shoot fresh weight **(D, I)**, and area of leaf #2 **(E, J)** in *Arabidopsis* seedlings subjected to control, Al, and Al+POT1 conditions (n=36, 4 plants pooled per data point, across three independent experiments). Boxplots depict upper and lower quartiles, while whiskers represent the range from the minimum to the lower quartile and from maximum to upper quartiles. Statistical significance of Al+POT1-treatment compared to control and Al-treatment was determined using Student’s *t*-test (*** *p* < 0.001, ** *p* < 0.01, * *p* < 0.05). Detailed *p* values are provided in **Supplementary Table 3**. Confocal laser scanning microscopy images depict morin staining for Al **(K)** and aniline blue staining for callose **(L)** in root tips of seedlings grown at pH 4. Scale bars: 100µm.

Seedlings grown on pH 4 and pH 5.6 agar plates with 100µM AlCl_3_ (Al-treated) or 100µM AlCl_3_+POT1 (Al+POT1-treated) for ten days displayed significant differences. At pH 4, Al-treated seedlings exhibited inhibited primary root growth, while Al+POT1-treated seedlings had longer primary roots, higher root and shoot fresh weights, and increased leaf area (**Figures 2A–E**). Total Al content in Al+POT1-treated roots and shoots was substantially lower than in Al-treated plants (**Supplementary Figure 2**). At pH 5.6, where Al is non-toxic, Al treatment had a less pronounced effect (**Figures 2F-J**). However, primary root lengths of Al+POT1-treated seedlings were consistently higher than control and Al-treated seedlings at both pH levels (**Figure 2**). Likewise, the shoot-to-root (S/R) ratio in Al+POT1-treated plants exceeded that in Al-treated plants at pH 4 and 5.6 (**Supplementary Figure 3**). Root staining revealed intense Al- and callose- specific signals in Al-treated roots but weak and diffused in control and Al+POT1-treated roots, indicating a reduction of Al accumulation and callose deposition at the root tips by POT1 (**Figures 2K, L**).

**Figure 3:**
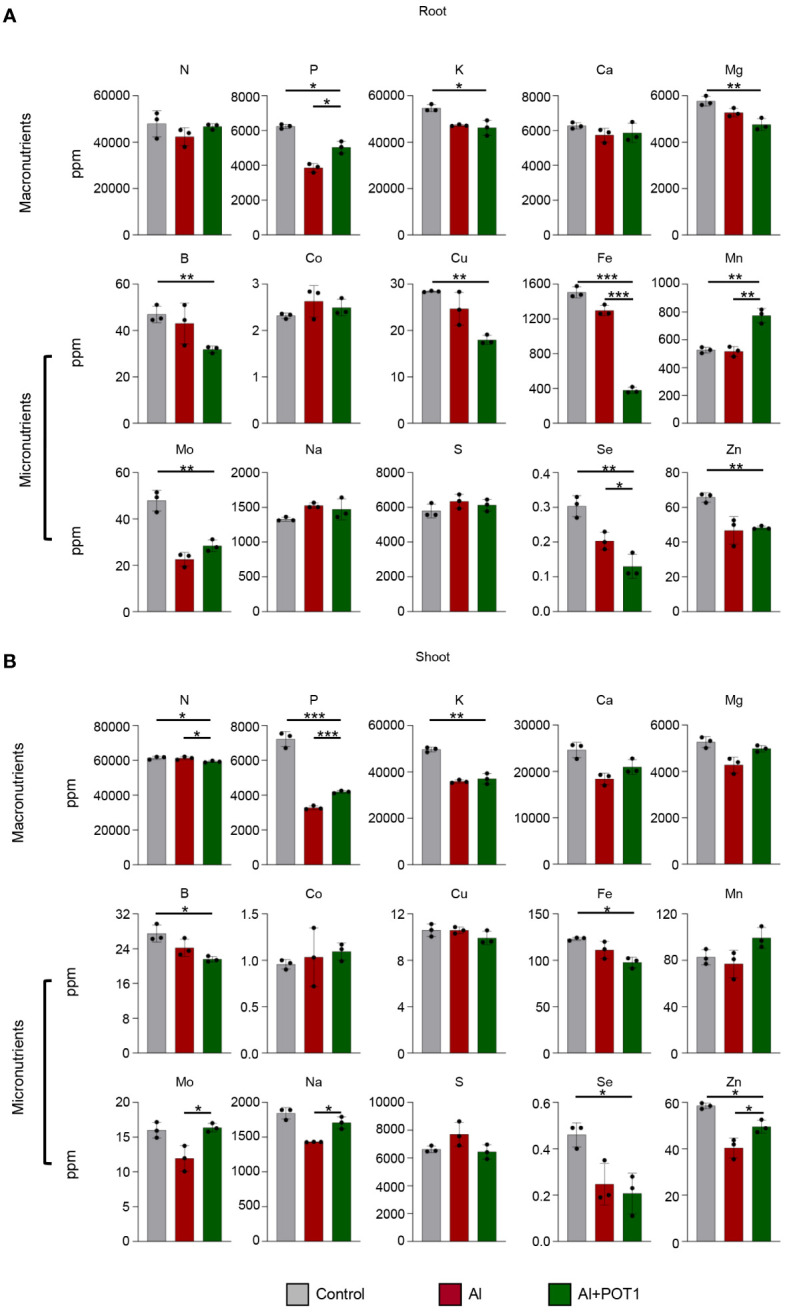
POT1 alters the nutrient levels in *Arabidopsis*. Analysis of macronutrient and micronutrient concentrations in the roots **(A)** and shoots **(B)** of *Arabidopsis* plants grown under control (0µM AlCl_3_), Al stress (100µM AlCl_3_), or Al stress with POT1 colonies (Al+POT1) at pH 4. Columns represent mean ± SD (more than 20 plants pooled per data point, across three independent experiments). Statistical significance of Al+POT1 compared to control and Al-treatment was determined using Student’s *t*-test (*** *p* < 0.001, ** *p* < 0.01, * *p* < 0.05). Detailed *p* values are provided in **Supplementary Table 6**.

In plants treated with Al+POT1, root analysis revealed elevated levels of nitrogen (N), phosphorus (P), carbon (C), hydrogen (H), manganese (Mn), and molybdenum (Mo) compared to those treated with Al alone (**Figure 3**; **Supplementary Figure 4**). Shoot analysis showed increased concentrations of macronutrients [P, calcium (Ca), magnesium (Mg)], micronutrients [Mn, Mo, sodium (Na), zinc (Zn)] and a higher carbon-to-nitrogen (C/N) ratio in Al+POT1-treated plants (**Figure 3**; **Supplementary Figure 4**). Conversely, Al+POT1-treated plants exhibited lower levels of iron (Fe) and selenium (Se) compared to Al-treated plants (**Figure 3**), highlighting the selective impact of POT1 on nutrient dynamics. These changes suggest that POT1 treatment may influence nutrient uptake and distribution, affecting specific nutrient translocation processes in response to Al stress.

### 3.2 POT1 boosts growth, reduces immune responses, suppresses Al-resistance genes, and changes nutrient transportation in plants under Al stress

To assess the impact of POT1 on plants under Al stress, we analyzed the transcriptomes of shoot and root in *Arabidopsis* seedlings treated with Al alone or with POT1. We obtained 154Gb of clean reads (12.83Gb/sample), with high sequencing quality (Q20 > 97%, Q30 > 92%). And a mapping rate averaging 97.7%. Expression levels were consistent, mostly around log_2_(FPKM + 1) values of 2.5. (**Supplementary Figure 5**; **Supplementary Tables 7**, **8**). Principal component analysis effectively segregated the four transcriptomes. Comparison of Al-treated shoots versus Al+POT1-treated shoots revealed 566 downregulated and 419 upregulated genes. Similarly, in the Al-treated roots versus Al+POT1-treated roots comparison, 512 downregulated and 483 upregulated genes were identified (**Supplementary Figure 5**).

**Supplementary Figure 6** illustrates enriched Gene Ontology (GO) terms and the protein-protein interaction (PPI) network among upregulated DEGs in Al+POT1-treated roots. These DEGs encompassed defense response to fungus, hydrogen peroxide/ROS metabolic processes, and phosphate ion transport. Conversely, downregulated DEGs in Al+POT1-treated roots were associated with OA and sulfate transport, water deprivation response, cytokinin metabolism and triterpenoid metabolism, with notable downregulation observed in genes related to lateral root initiation (**Supplementary Figure 7**). Notably, the downregulation of C-terminally encoded peptide 5 (*CEP5*), known for repressing primary root length and lateral root initiation (Roberts et al., 2016), was observed in the roots of Al+POT1-treated plants (**Supplementary Figure 7**). For Al+POT1-treated shoots, GO and PPI analysis of upregulated DEGs revealed enrichment in phosphate starvation response, phosphatidylinositol phosphate biosynthesis, and seed germination. alongside genes linked to organ growth such as *NGA3*, *ORS1*, *ARGOS*, *TCP1*, *HAT5*, and *KAN1*, including the negative senescence regulator, *ESP* (**Supplementary Figure 8**). On the other hand, downregulated DEGs in Al+POT1-treated shoots were predominately associated with immune response, with additional connections to protein folding and degradation, oxidative stress response, and seed storage (**Supplementary Figure 9**).

In Al+POT1-treated roots, genes associated with OA exudation and Al sequestration like *ALMT1*, *MATE*, *ALS1*, *ALS3*, and *NIP1–2* were downregulated, while *STOP1* remained unchanged and *RAE1* and *RAH1* were slightly upregulated. *ESD4* and *HPR1*, involved in STOP1 regulation, were downregulated (**Table 1**). Additionally, callose accumulation genes remained mostly unchanged, while glucan endo-1,3-beta-glucosidases, the callose degradation-related genes, were upregulated, and genes encoding callose-binding proteins PDCB3–5 were downregulated in Al+POT1-treated roots (**Supplementary Table 10**).

**Table 1:** Expression levels of plant genes related to Al-resistance in roots (Al-R) and shoots (Al-S) of Al-treated plants and in roots (Al+POT1-R) and shoots (Al+POT1-S) of Al-treated plants with POT1 inoculation.

Gene ID	Gene Name	Gene description	Al+POT1-R vs Al-R		Al+POT1-S vs Al-S	
			log_2_FC	*p* value	log_2_FC	*p* value
AT1G08430	*ALMT1*	Aluminum-activated malate transporter 1	-0.074	0.0095	0.176	0.0094
AT1G51340	*MATE*	Protein Detoxification	-0.312	0.0002	-0.358	0.0006
AT1G34370	*STOP1*	Protein Sensitive to Proton Rhizotoxicity1	0.146	0.0140	0.063	0.0614
AT4G15880	*ESD4*	Ubiquitin-like-specific protease	-0.132	0.0234	0.053	0.0731
AT5G09860	*HPR1*	Hyperrecombination protein 1, a subunit of THO/TREX complex	-0.098	0.0489	0.081	0.0503
AT5G01720	*RAE1*	Regulation of *ALMT1* expression 1	0.476	0.0059	0.132	0.0580
AT5G27920	*RAH1*	*RAE1* homolog 1	0.435	0.0051	0.086	0.0054
AT5G39040	*ALS1*	ABC transporter, aluminum-sensitive 1	0.136	0.0254	-0.161	0.0498
AT2G37330	*ALS3*	ABC transporter-like protein	-0.548	0.0049	0.016	0.0918
AT4G18910	*NIP1–2*	Al-malate transporter	-0.211	0.0428	0.017	0.0894

In Al+POT1-treated roots, nutrient transporter genes for Cu, P, Na, and Mo were upregulated, particularly phosphate transporters (*PHT1–3*, *PHT1–2*, *PHO1-H1*, *PHT1–9*, *PHT1–1*, *PHO1*). However, sulfate transporters were downregulated. In Al+POT1-treated shoots, *PHO1-H10* showed moderate upregulation. Notably, genes encoding MATE efflux family proteins and heavy metal transport/detoxification superfamily proteins were downregulated in both roots and shoots of Al+POT1-treated plants compared to Al-treated ones (**Supplementary Table 11**).

### 3.3 POT1 promotes plant growth in Al-rich soils and in acidic soils

**Figure 4:**
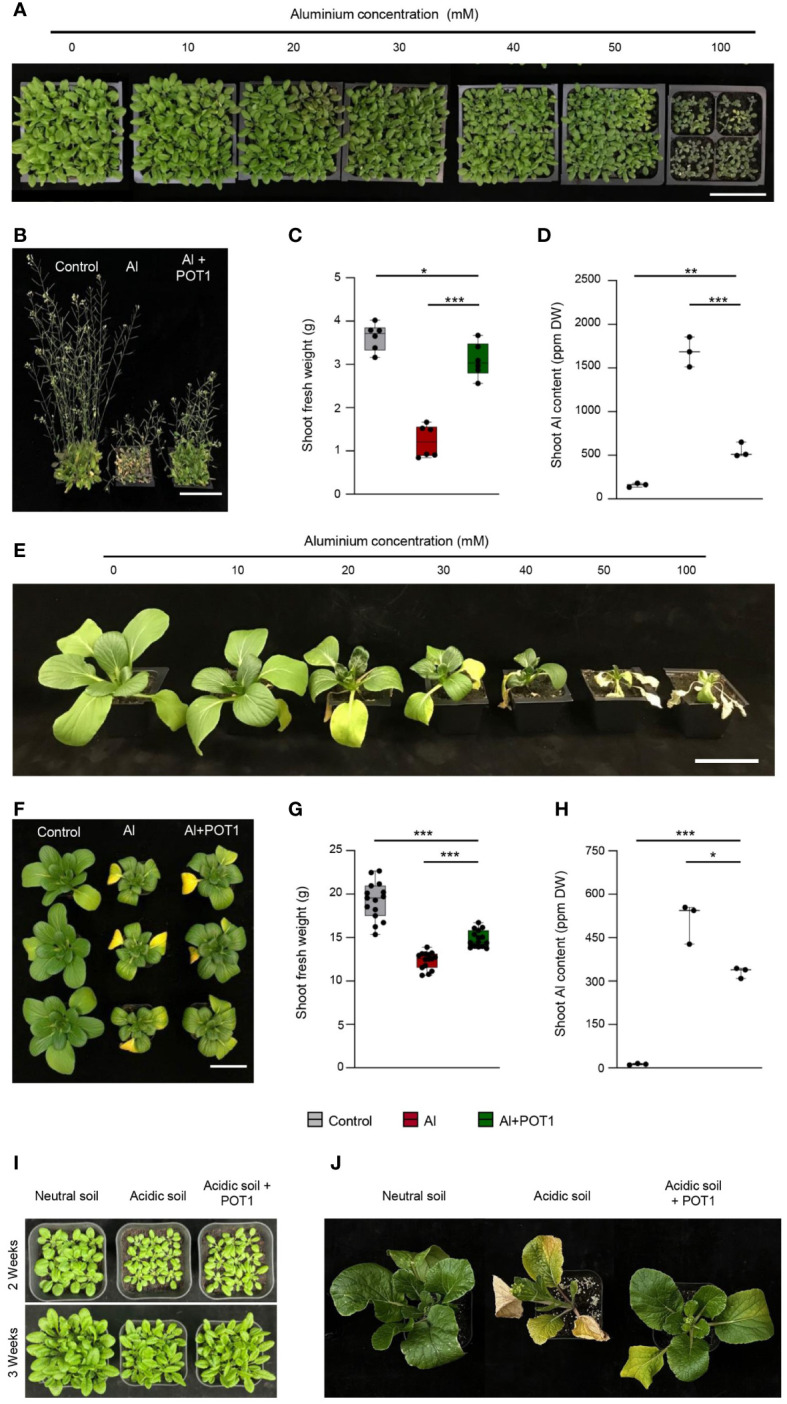
POT1 improves plant growth in Al-rich and acidic soils. Growth assessment of *Arabidopsis* **(A)** and Pak Choi **(E)** plants exposed to varying concentrations of AlCl_3_ (10, 20, 30, 40, 50, and 100mM) relative to the control (0mM). Scale bar: 10cm. Representative images of *Arabidopsis* **(B)** and Pak Choi **(F)** plants subjected to different treatments: control (0mM AlCl_3_), Al stress (50mM AlCl_3_ for *Arabidopsis* and 20mM AlCl_3_ for Pak Choi), or Al stress with POT1 inoculation (Al+POT1). Scale bar: 10cm. Comparison of shoot fresh weight **(C, G)** and shoot Al content **(D, H)** in *Arabidopsis* and Pak Choi plants, respectively. For *Arabidopsis* shoot fresh weight, data were pooled from 54 plants across three independent experiments, with 9 plants per data point. *Arabidopsis* shoot Al content data were aggregated from over 20 plants per data point across three independent experiments. For Pak Choi shoot fresh weight, data were collected from 15 plants across three independent experiments. Pak Choi shoot Al content data were pooled from more than 3 plants per data point across three independent experiments. Boxplots depict upper and lower quartiles, while whiskers represent the range from the minimum to the lower quartile and from maximum to upper quartiles. Statistical significance of Al+POT1-treatment compared to control and Al-treatment was determined using Student’s *t*-test (*** *p* < 0.001, ** *p* < 0.01, * *p* < 0.05). Detailed *p* values are provided in **Supplementary Table 12**. Representative images of *Arabidopsis* **(I)** and Choy Sum **(J)** plants cultivated in neutral soil (pH 5.6), acidic soil (pH 4), and acidic soil with POT1 inoculation (acidic soil + POT1).

One-week-old seedlings of *Arabidopsis* and Pak Choi were transplanted into soil and subjected to different concentrations (0–100mM) of AlCl_3_ solution weekly. As the Al concentration in the soil increased, *Arabidopsis* showed gradual reduction in size, with severe stunting and purple discoloration observed at 100mM Al, indicating anthocyanin accumulation (**Figure 4A**). Significant effects on Pak Choi plants were observed at 30mM Al (**Figure 4E**). Based on these findings, effective concentrations of 50mM Al for *Arabidopsis* and 20mM Al for Pak Choi were selected to examine the role of POT1 in Al toxicity.

In *Arabidopsis* treated with Al, the plants exhibited smaller leaves and shorter height, and their senescence occurred earlier compared to those treated with Al+POT1 (**Figure 4B**). Al+POT1-treated plants exhibited a 3-fold increase in shoot fresh weight and a 2-fold increase in chlorophyll content compared to Al-treated plants (**Figure 4C**; **Supplementary Figure 10A**), along with a less stressed phenotype showing a 2-fold decrease in anthocyanin content (**Supplementary Figure 10B**). Analysis of Al content revealed that Al+POT1-treated plants contained approximately 500mg/kg Al in shoots, nearly 70% lower than the ~1700mg/kg in Al-treated plants (**Figure 4D**). Similarly, Pak Choi plants treated with Al+POT1 exhibited delayed senescence, higher shoot fresh weight, and lower shoot Al content compared to Al-treated plants (**Figures 4F-H**). POT1 also functions in remedying soil pH to enhance plant growth. While the *Arabidopsis* and Pak Choi plants cultivated in acidic soil (pH 4) displayed stunted growth compared to those grown in neutral soil (pH 5.6). Inoculating acidic soil with POT1 one week prior to seedling transplantation resulted in improved growth of these plants, indicating that POT1 enhances plant growth and resilience (**Figures 4I, J**).

### 3.4 POT1 has superior Al stress alleviation compared to other plant-growth-promoting *Penicillium* species

**Figure 5:**
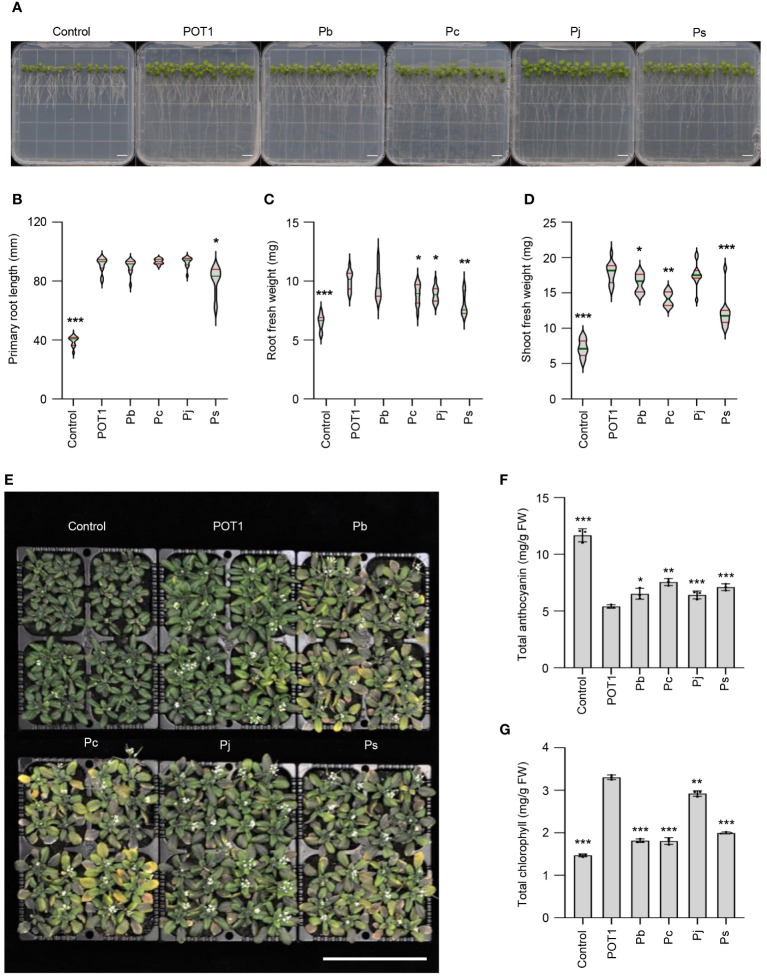
POT1 exhibits superior alleviation of Al stress compared to other *Penicillium* species. **(A)** *Arabidopsis* seedlings grown on agar plates under Al stress conditions with or without *Penicillium* strains. Scale bar: 1cm. **(B-D)** Comparison of primary root length **(B)**, root fresh weight **(C)**, and shoot fresh weight **(D)** of *Arabidopsis* seedlings with or without *Penicillium* strains under Al stress (n=12, across three independent experiments). Red lines represent quartiles, and green lines depict the median. **(E)** *Arabidopsis* seedlings grown on soil under control conditions and with 100mM AlCl_3_ without or with *Penicillium* species. Scale bar: 10cm. **(F, G)** Comparison of total anthocyanin **(F)** and total chlorophyll **(G)** content in *Arabidopsis* plants grown on soil under Al stress with or without *Penicillium* species (n=9, across three independent experiments). The abbreviations used are POT1 for *Penicillium olsonii* TLL1, Pb for *Penicillium bilaiae*, Pc for *Penicillium chrysogenum*, Pj for *Penicillium janthinellum*, Ps for *Penicillium simplicissimum.* Statistical significance of the POT1 treatment compared to control and other fungal treatments was determined using Student’s *t*-test (* *p* < 0.05, ** *p* < 0.01, *** *p* < 0.001). Detailed *p* values are provided in **Supplementary Table 14**.

In a comparative study, POT1 along with four other *Penicillium* species (*Penicillium bilaiae*, *Penicillium chrysogenum*, *Penicillium janthinellum*, and *Penicillium simplicissimum*) were subjected to varying concentrations of Al. Results revealed that *P. janthinellum* and *P. simplicissimum* exhibited the highest Al tolerance (>20mM), followed by *P. bilaiae* and POT1 (5mM), while *P. chrysogenum* had the lowest Al tolerance (**Supplementary Figure 11**). Subsequently, *Arabidopsis* plants were exposed to high Al conditions along with these *Penicillium* species to evaluate their ability to alleviate Al-stress. Plants inoculated with fungus, particularly POT1, displayed longer primary roots and higher root- and shoot-fresh weights compared to uninoculated controls (**Figures 5A–D**). Notably, POT1-inoculated plants outperformed others in both root and shoot fresh weights. When *Arabidopsis* was grown under high Al conditions in soil either with or without inoculation with these *Penicillium* species, POT1-inoculated plants exhibited the lowest accumulation of anthocyanin and the highest chlorophyll content (**Figures 5E–G**). These findings underscore the superior ability of POT1 to mitigate Al stress compared to other *Penicillium* species known for promoting plant growth.

## 4 Discussion

In our study, *Penicillium olsonii* TLL1 (POT1) tolerated up to 5mM Al and showed pH tolerance from 2 to 11, making it suitable for acidic soils (**Figure 1**). ICP and GC-MS analyses of POT1 suggested that both internal and external sequestration of Al^3+^ may play a role in its tolerance (**Figure 1**). These mechanisms are consistent with those reported in other Al-tolerant fungi (Kochian, 1995; Zhang et al., 2002; Yang et al., 2012). Al exposure triggers ROS accumulation, leading to lipid peroxidation, organelle dysfunction, and cellular damage, hindering root elongation (Liu et al., 2022). Analysis revealed upregulation of genes associated with antioxidative activity and ROS detoxification in Al+POT1-treated roots and shoots (**Supplementary Figures 6**, **8**), which is also consistent with previous findings showing increased expression of antioxidative enzymes improving Al tolerance in plants (Huang et al., 2021).

Plants respond to Al stress by accumulating high Al levels in root tips and translocating it to shoots for vacuolar sequestration (Kochian et al., 2015). However, reduced Al uptake by Al+POT1-treated plants compared to Al-treated plants (**Figure 2K**; **Supplementary Figure 2**) showed that the Al tolerance imparted by POT1 is different from that of *P. janthinellum* LK5, which helped plants in extracting and translocating higher Al in shoots and roots of tomato plants (Khan et al., 2015). Similarly, downregulation of well-known Al-stress responsive genes (*ALMT1*, *MATE*, *ALS3* and *NIP1–2*) in Al+POT1-treated roots compared to Al-treated roots (**Table 1**) is contrary to the result where Al-resistant, plant growth promoting bacterial strains induced overexpression of these to increase tolerance of ginseng plants against Al stress (Farh et al., 2017). Callose deposition at the root tips is a hallmark symptom of Al toxicity (Sivaguru and Horst, 1998; Sivaguru et al., 2000). The decreased deposition of callose observed in the roots of plants treated with Al+POT1 (**Figure 2L**) could be attributed to the heightened breakdown of callose, possibly facilitated by the upregulation of *Arabidopsis* β-1,3-glucanase *BG1* expression, as indicated in **Supplementary Table 10**.

Reduction in root biomass and the extensive root injury caused by Al leads to poor uptake of nutrients (Kochian, 1995). But, under high Al conditions, POT1 treatment upregulated the expression of the nutrient transporters in roots, resulting in improved nutrient levels in both roots and shoots compared to Al-treated plants (**Figure 3**; **Supplementary Figure 4**, **Supplementary Table 11**). Enhanced Mn uptake in POT1-inoculated plants could account for the elevated phosphorelay signal transduction and increased antioxidant activity, as Mn is a crucial co-factor for enzymes involved in photosynthesis and ROS scavenging (Schmidt and Husted, 2019). Interestingly, Fe uptake significantly differed between Al-treated roots (1200ppm) and Al+POT1-treated roots (400ppm). This was attributed to Fe unavailability in POT1-inoculated soil, as POT1 internalizes Fe in addition to Al in acidic soils (**Supplementary Figure 12**). Sequestration of Al and Fe, commonly present in toxic concentrations in acidic soils, by POT1 positions it as a potential asset for enhancing yields in such environments.

Al serves as a trigger for stress response pathways in plants like wheat (Houde and Diallo, 2008). In Al+POT1-inoculated plants, a decrease in the expression of disease-resistance and systemic acquired resistance genes was observed in shoots (Clusters 1 and 4; **Supplementary Figure 9**) along with the downregulation of genes (*BIP1*, *BIP2*, *CRT2*, *HRD1B*) involved in protein folding and degradation of misfolded/damaged proteins under stress conditions (Cluster 5; **Supplementary Figure 9**) indicating a potential reduction in Al stress levels compared to Al-treated shoots. While Al is recognized for triggering pathogenesis-related (*PR*) signaling pathways in plants (Hamel et al., 1998), the expression of *PR* genes in *Arabidopsis* roots showed no significant change despite the penetration and hyphae formation by POT1. Only minor induction was observed in shoots (**Supplementary Figure 13**).

**Figure 6:**
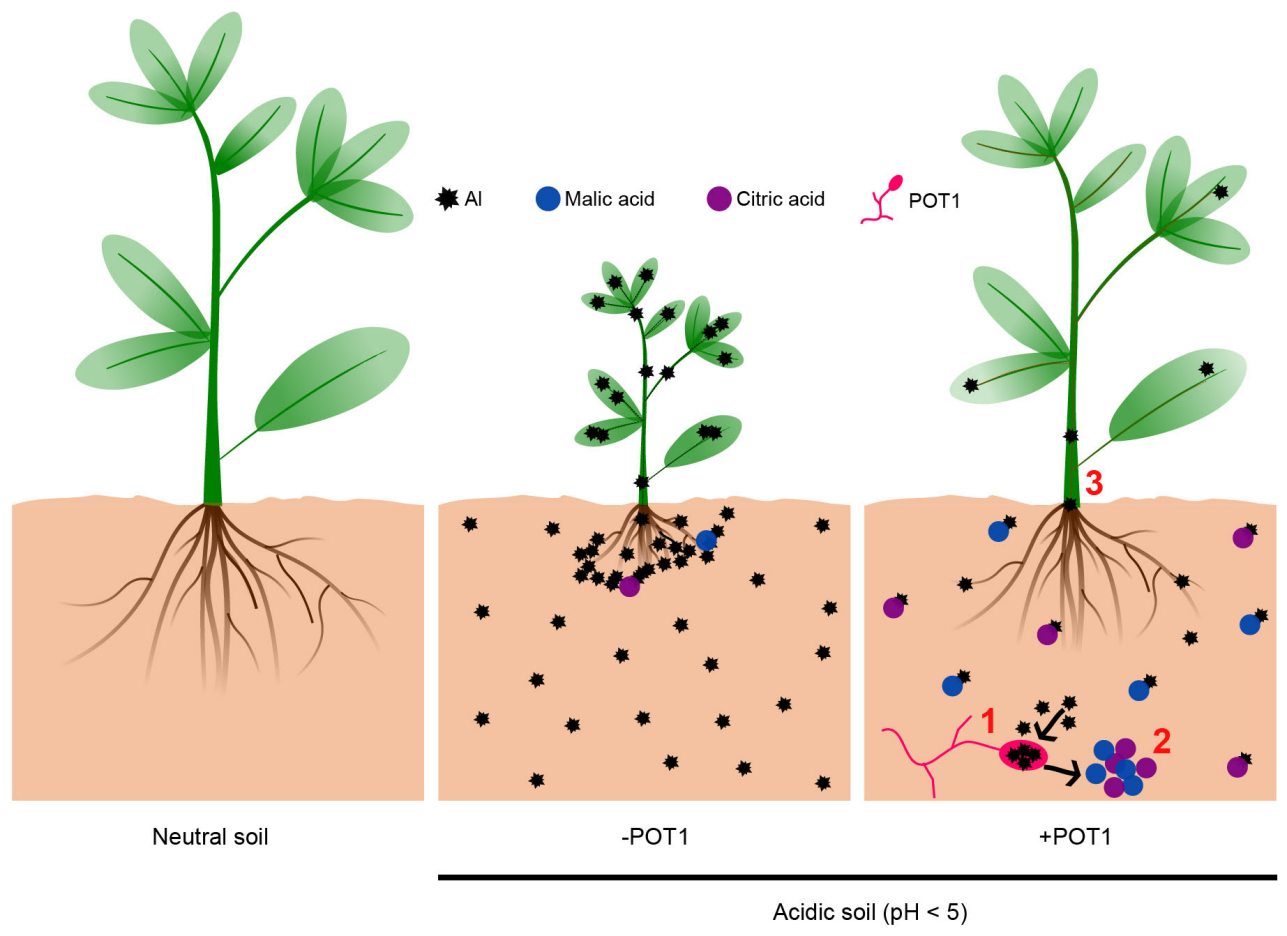
The mechanism by which *Penicillium olsonii* TLL1 alleviates Al-toxicity to promote plant growth. This simplified model demonstrates how POT1 enhances plant growth under Al stress in acidic soils through three key processes: 1. POT1 internalizes Al, thereby reducing its concentration in the soil. 2. In the presence of Al, POT1 produces malic and citric acids, likely serving as a detoxification mechanism. 3. Plants treated with POT1 show lower Al content in the roots and shoots compared to untreated plants.

In acidic soils, even plants like barley, known for their resilience, face growth challenges due to Al toxicity (Forster et al., 2000). Results obtained in this study suggests that POT1 promotes plant growth under Al-rich acidic conditions by counteracting Al toxicity through both internal sequestration of Al^3+^ and external detoxification of Al^3+^ via secretion and chelation of organic acids in the rhizosphere (**Figure 6**). These results, coupled with its superior performance under high Al conditions (**Figure 5**), make POT1 a promising candidate for incorporation into organic fertilizers. This approach could potentially serve as a strategy to overcome soil acidity, alleviate Al toxicity, boost plant biomass, and enhance fertility in acidic soils, particularly under changing climatic conditions. The introduction of POT1 can not only increase the yield of grains and leafy vegetables in unfavorable soil conditions but also contribute to the safety of these crops for consumption.

## 5 Conclusions

In conclusion, our study underscores the potential of POT1 as a promising solution for addressing Al toxicity in acidic soils. The remarkable tolerance of POT1 to high Al concentrations, along with its ability to enhance plant growth while reducing Al accumulation, highlights its potential as a biofortification agent. Comparative analysis confirmed superiority of POT1 over other Al-tolerant *Penicillium* species in alleviating Al toxicity. Given the prevalence of acid sulfate soils in Southeast Asia, the strong acid-alkali tolerance of POT1 suggests its viability for converting highly acidic soils into arable land. Further research should focus on understanding the interaction of POT1 with microbiomes, assessing its effectiveness across diverse soil textures, and exploring its ability to mitigate Al stress in rice, particularly in paddy soils. Additionally, investigating the efficacy of POT1 in combating other abiotic stresses induced by environmental changes like high temperatures or drought is crucial for its potential in sustainable crop production and resilience in the face of climate change.

## Data availability statement

The original contributions presented in the study are publicly available. This data can be found here: NCBI, PRJNA1047286.

## Author contributions

SD: Formal analysis, Investigation, Methodology, Project administration, Validation, Visualization, Writing – original draft, Writing – review & editing. YS: Formal analysis, Investigation, Writing – original draft. VA: Formal analysis, Investigation, Writing – original draft. ES: Investigation, Writing – original draft. BP: Conceptualization, Funding acquisition, Methodology, Project administration, Supervision, Writing – original draft, Writing – review & editing.
