# Supplementary material for: Mitigating aluminum toxicity and promoting plant resilience in acidic soil with Penicillium olsonii TLL1
Source: Front Plant Sci. 2024 Jun 20;15:1423617. doi: 10.3389/fpls.2024.1423617 (PMC11225409; doi:10.3389/fpls.2024.1423617)
Supplement: Supplementary file 1 [file DataSheet_1.docx]

Supplementary Material

**Mitigating aluminum toxicity and promoting plant resilience in acidic soil with *Penicillium olsonii* TLL1**

Savitha Dhandapani, Yee Hwui Sng, Valiya Nadakkakath Agisha, Erinjery Jose Suraby, Bong Soo Park^*^

* Corresponding author

Bong Soo Park

E-mail: bongsoo@tll.org.sg

**This file includes:**

Supplementary Figures 1 to 13

Supplementary Tables 1 to 16

**
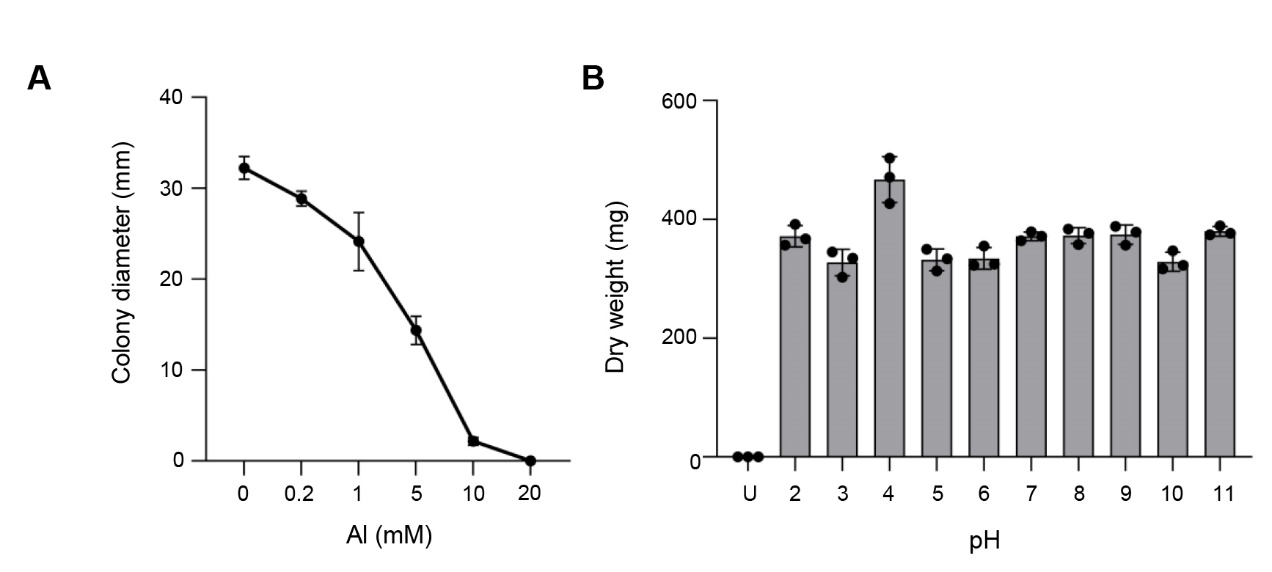
**

**Supplementary Figure 1.** POT1 has Al and pH tolerance. (A) Colony diameter measurements of POT1 on malt extract agar supplemented with varying concentrations of AlCl_3_ (0.2-20mM) compared to the control (0mM). (B) Determination of dry weight of POT1 in malt extract broth at pH levels ranging from 2 to 11, illustrating its pH tolerance. "U" denotes uninoculated plain medium. Columns represent mean ± SD of three independent experiments.

**
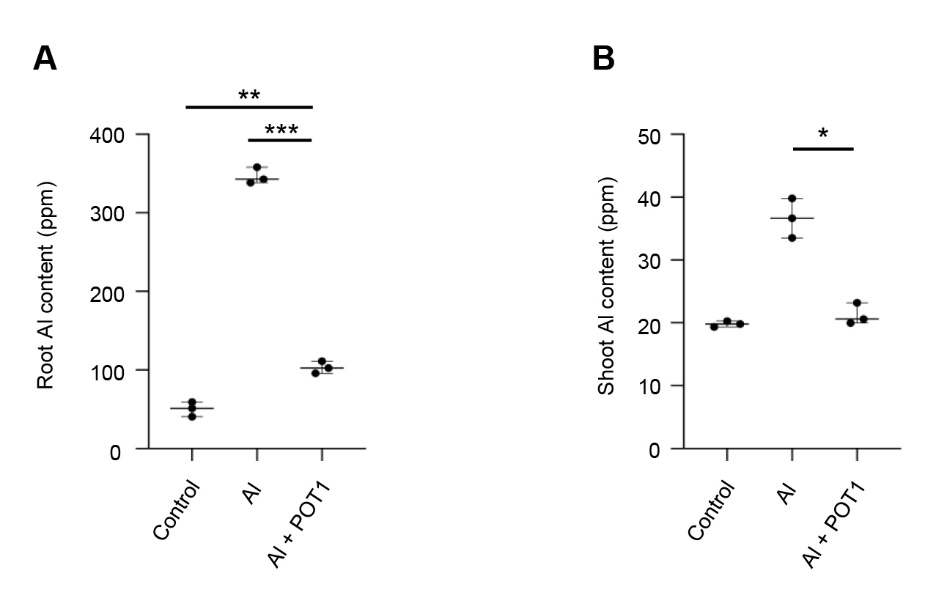
**

**Supplementary Figure 2.** POT1 reduces Al content in *Arabidopsis* roots and shoots. Analysis of Al content in the roots (A) and shoots (B) of *Arabidopsis* seedlings subjected to different treatments: control (0µM AlCl_3_), Al stress (100µM AlCl_3_), or Al stress with POT1 colonies (Al+POT1) at pH 4 (based on more than 20 plants pooled per data point, across three independent experiments). Boxplots depict upper and lower quartiles, while whiskers represent the range from the minimum to the lower quartile and from maximum to upper quartiles. Statistical significance of Al+POT1-treatment compared to control and Al-treatment was determined using Student’s *t*-test (*** *p* < 0.001, ** *p* < 0.01, * *p* < 0.05). Detailed *p* values are provided in Supplementary Table 4.

**
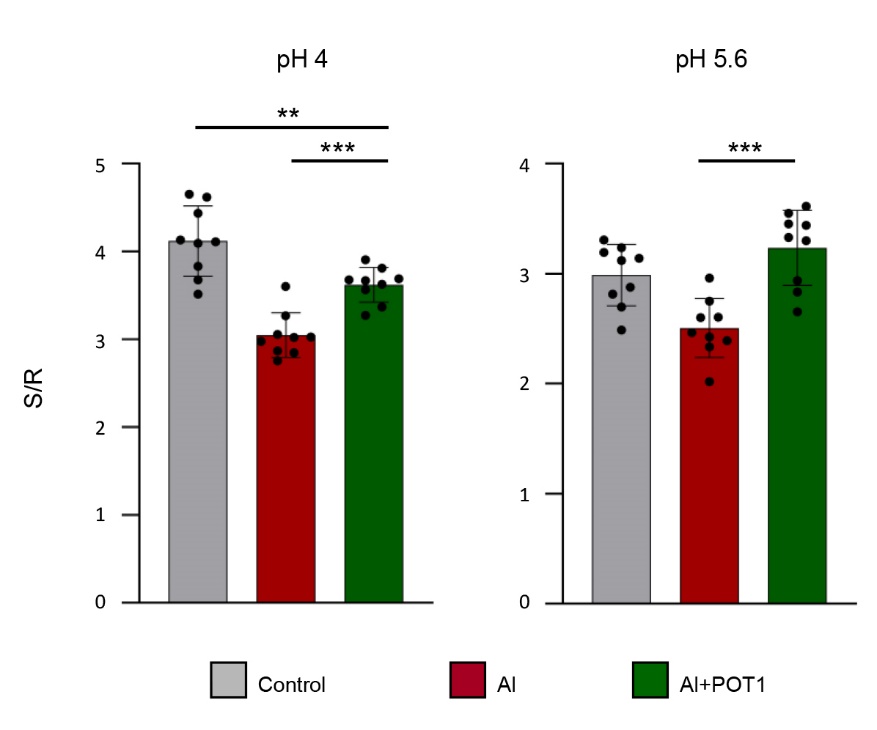
**

**Supplementary Figure 3.** POT1 enhances the shoot-to-root ratio in *Arabidopsis* seedlings. Analysis of the **s**hoot-to-root ratio (S/R) of *Arabidopsis* seedlings subjected to different treatments: control (0µM AlCl_3_), Al stress (100µM AlCl_3_), or Al stress with POT1 colonies (Al+POT1) at pH 4 and pH 5.6. Columns represent mean ± SD (n=36, 4 plants pooled per data point, across three independent experiments). Statistical significance of Al+POT1-treatment compared to control and Al-treatment was determined using Student’s *t*-test (*** *p* < 0.001, ** *p* < 0.01). Detailed *p* values are provided in Supplementary Table 5.

**
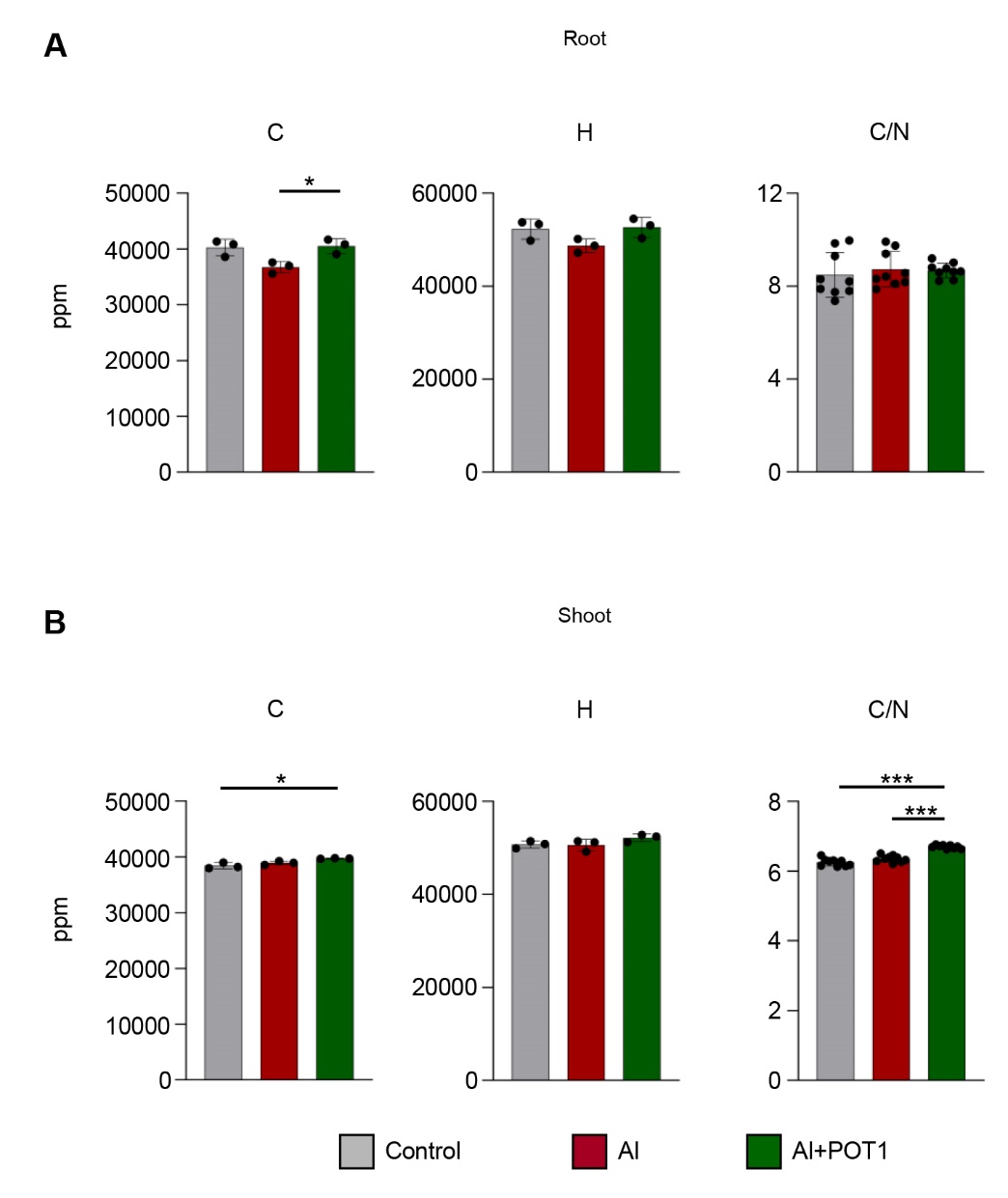
**

**Supplementary Figure 4.** POT1 increases carbon, hydrogen levels, and alters the carbon-to-nitrogen ratio. Analysis of carbon (C) and hydrogen (H) content, along with the carbon-to-nitrogen (C/N) ratio in the roots (A) and shoots (B) of Arabidopsis plants subjected to different treatments: control (0µM AlCl_3_), Al stress (100µM AlCl_3_), or Al stress with POT1 colonies (Al+POT1) at pH 4. Columns represent mean ± SD (based on more than 20 plants per data point, across three independent experiments). Statistical significance of Al+POT1-treatment compared to control and Al-treatment was determined using Student’s *t*-test (*** *p* < 0.001, * *p* < 0.05). Detailed *p* values are provided in Supplementary Table 7.

**
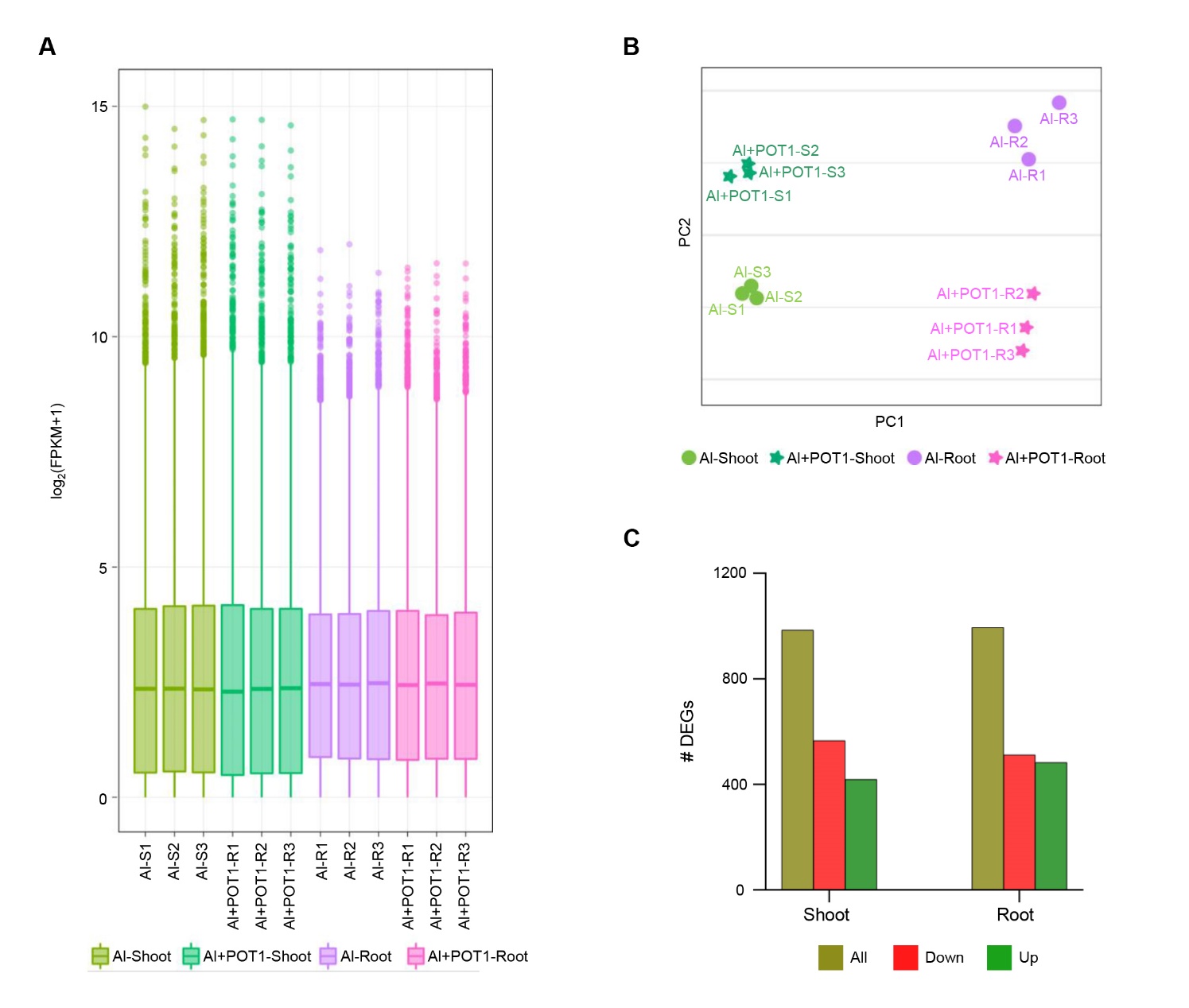
**

**Supplementary Figure 5.** Overview of transcriptome analysis comparing Al-treated and Al+POT1-treated plants. (A) Distribution of gene expression levels in Al-treated and Al+POT1-treated plants depicted through box plots of FPKM values. The boxes represent quartiles, whiskers display the range from minimum to lower quartile and from maximum to upper quartiles, and solid lines indicate average log_2_(FPKM+1) values. (B) Principal component analysis (PCA) of RNA-seq samples, where each point represents a sample. Samples with similar gene expression patterns cluster together, with different treatments and tissues color-coded. (C) Differentially expressed genes (DEGs) identified in shoots and roots of Al-treated and Al+POT1-treated plants through RNA-seq analysis.

**
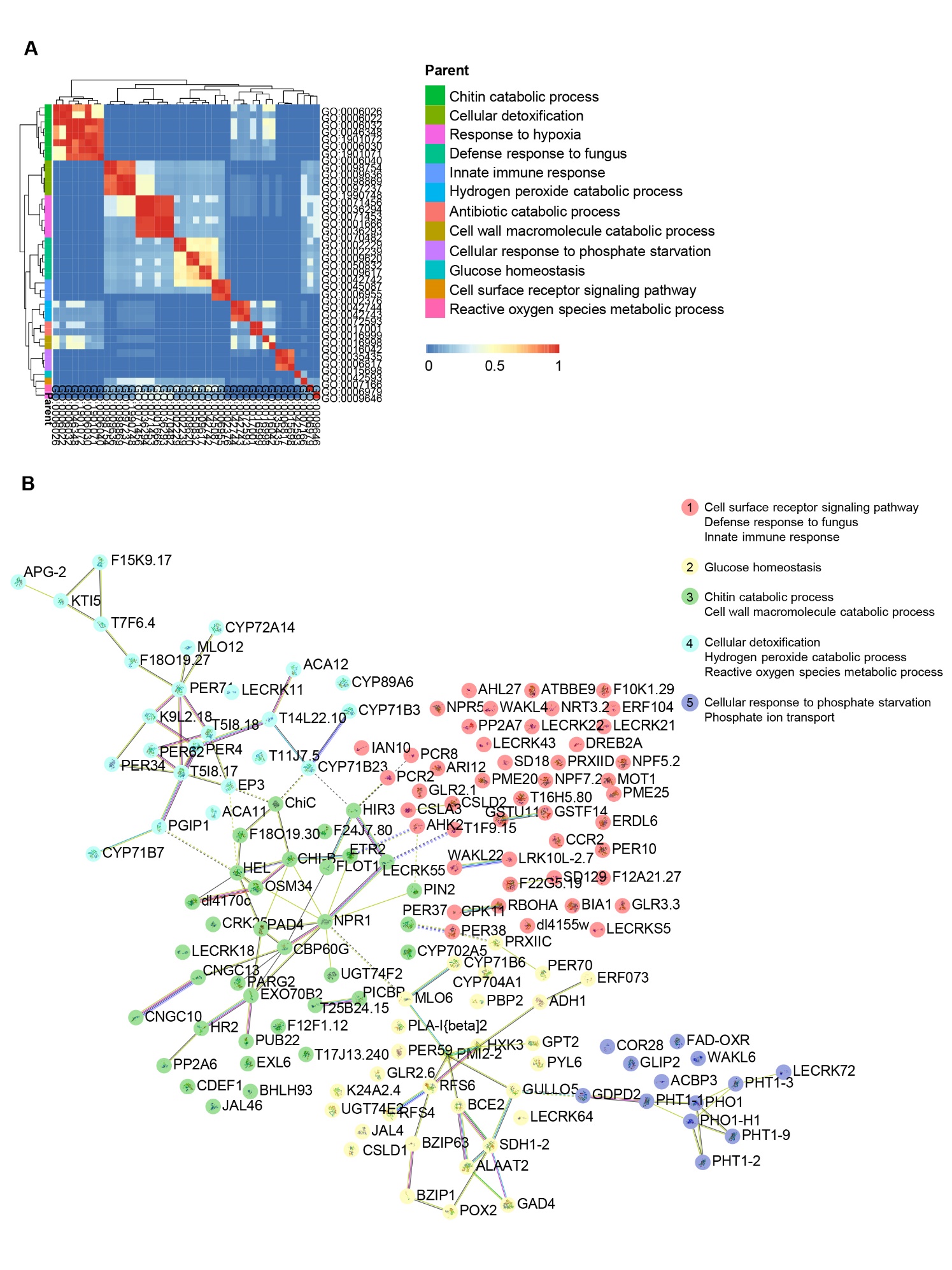
**

**Supplementary Figure 6.** Integrated analysis of enriched biological processes and protein-protein interactions among upregulated genes in the roots of Al+POT1-treated plants. (A) Gene Ontology (GO) analysis highlighting enriched biological processes. (B) Mapping of differentially up-regulated genes to the STRING database, revealing significant interactions (score >0.4). Nodes represent proteins, and connecting lines indicate specific protein-protein associations contributing to shared functions, not necessarily physical binding. Dotted lines connect distinct clusters. Line colors signify the type of interaction evidence: cornflower blue for known interactions from curated databases, purple for experimentally determined interactions, green for predictions based on gene neighborhood, red for predicted interactions from gene fusions, navy blue for predictions from gene co-occurrence, light green for predictions from text mining, olympic-blue for predictions from protein homology, and black for predictions based on co-expression. The network was further segmented into five specified clusters using kmeans clustering.

**
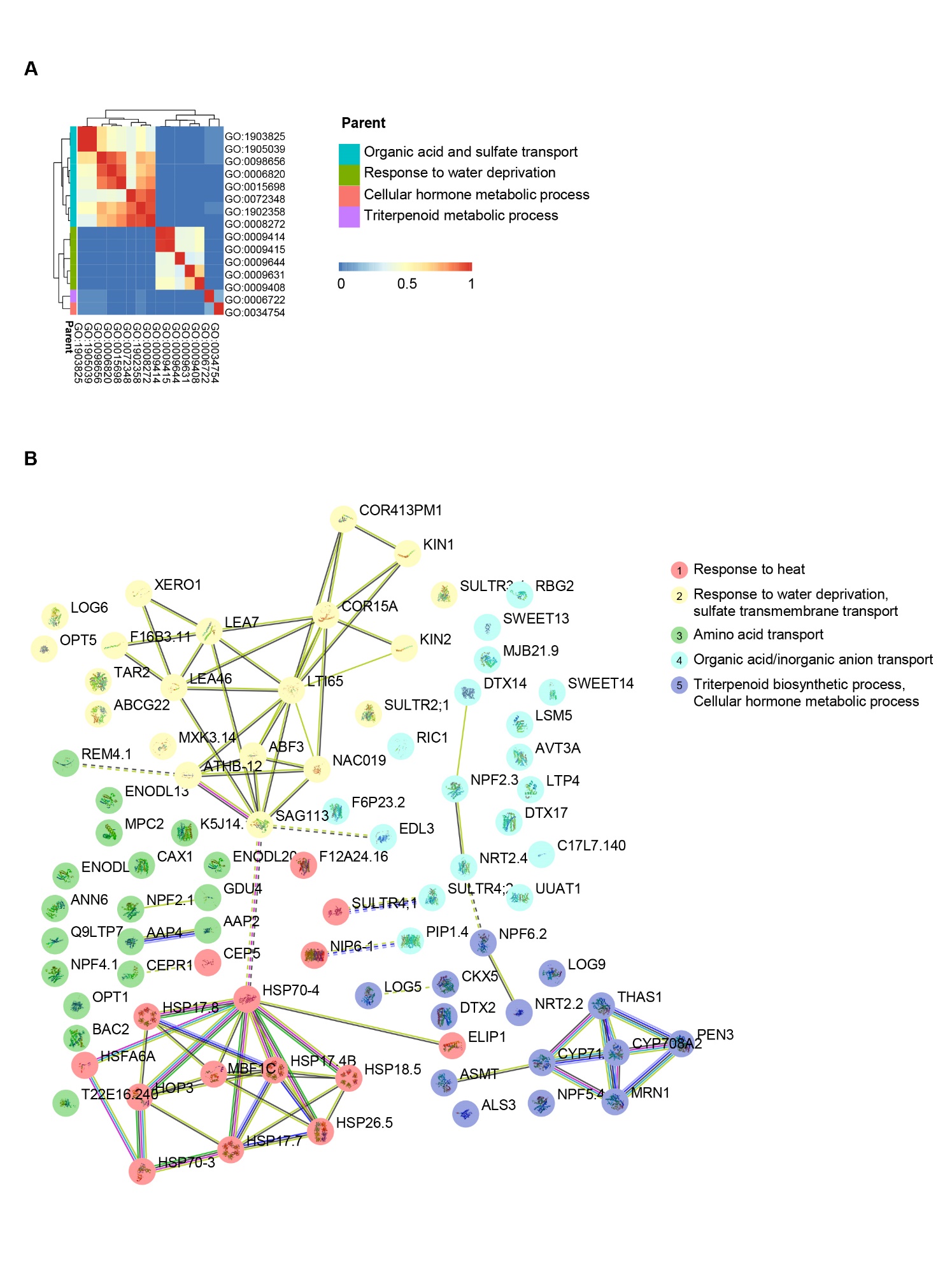
**

**Supplementary Figure 7.** Integrated analysis of enriched biological processes and protein-protein interactions among downregulated genes in the roots of Al+POT1-treated plants. (A) Gene Ontology (GO) analysis highlighting enriched biological processes. (B) Mapping of differentially down-regulated genes to the STRING database, revealing significant interactions (score >0.4). Nodes represent proteins, and connecting lines indicate specific protein-protein associations contributing to shared functions, not necessarily physical binding. Dotted lines connect distinct clusters. Line colors signify the type of interaction evidence: cornflower blue for known interactions from curated databases, purple for experimentally determined interactions, green for predictions based on gene neighborhood, red for predicted interactions from gene fusions, navy blue for predictions from gene co-occurrence, light green for predictions from text mining, olympic-blue for predictions from protein homology, and black for predictions based on co-expression. The network was further segmented into five specified clusters using kmeans clustering.

**
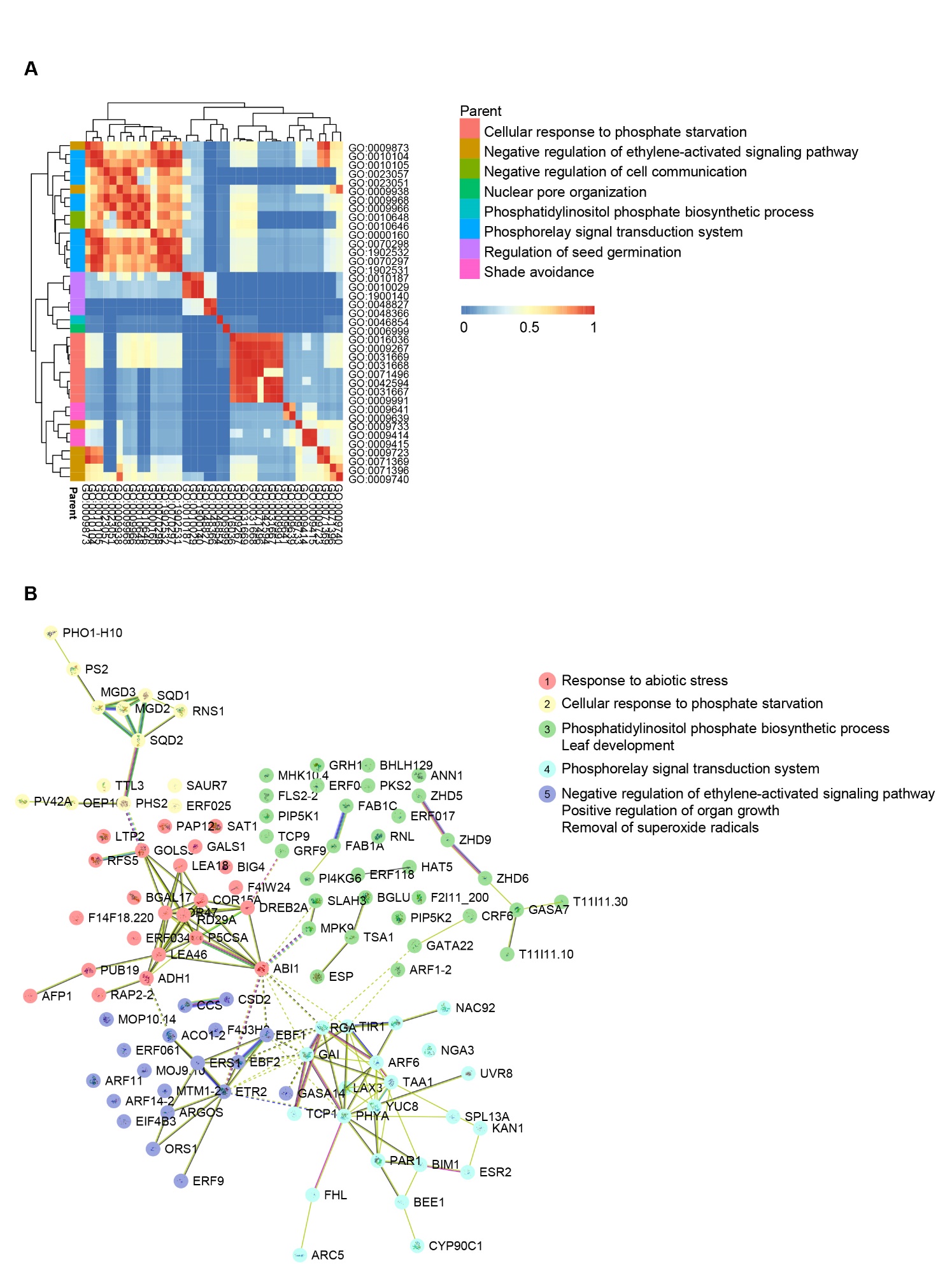
**

**Supplementary Figure 8.** Integrated analysis of enriched biological processes and protein-protein interactions among upregulated genes in the shoots of Al+POT1-treated plants. (A) Gene Ontology (GO) analysis highlighting enriched biological processes. (B) Mapping of differentially up-regulated genes to the STRING database, revealing significant interactions (score >0.4). Nodes represent proteins, and connecting lines indicate specific protein-protein associations contributing to shared functions, not necessarily physical binding. Dotted lines connect distinct clusters. Line colors signify the type of interaction evidence: cornflower blue for known interactions from curated databases, purple for experimentally determined interactions, green for predictions based on gene neighborhood, red for predicted interactions from gene fusions, navy blue for predictions from gene co-occurrence, light green for predictions from text mining, olympic-blue for predictions from protein homology, and black for predictions based on co-expression. The network was further segmented into five specified clusters using kmeans clustering.

**
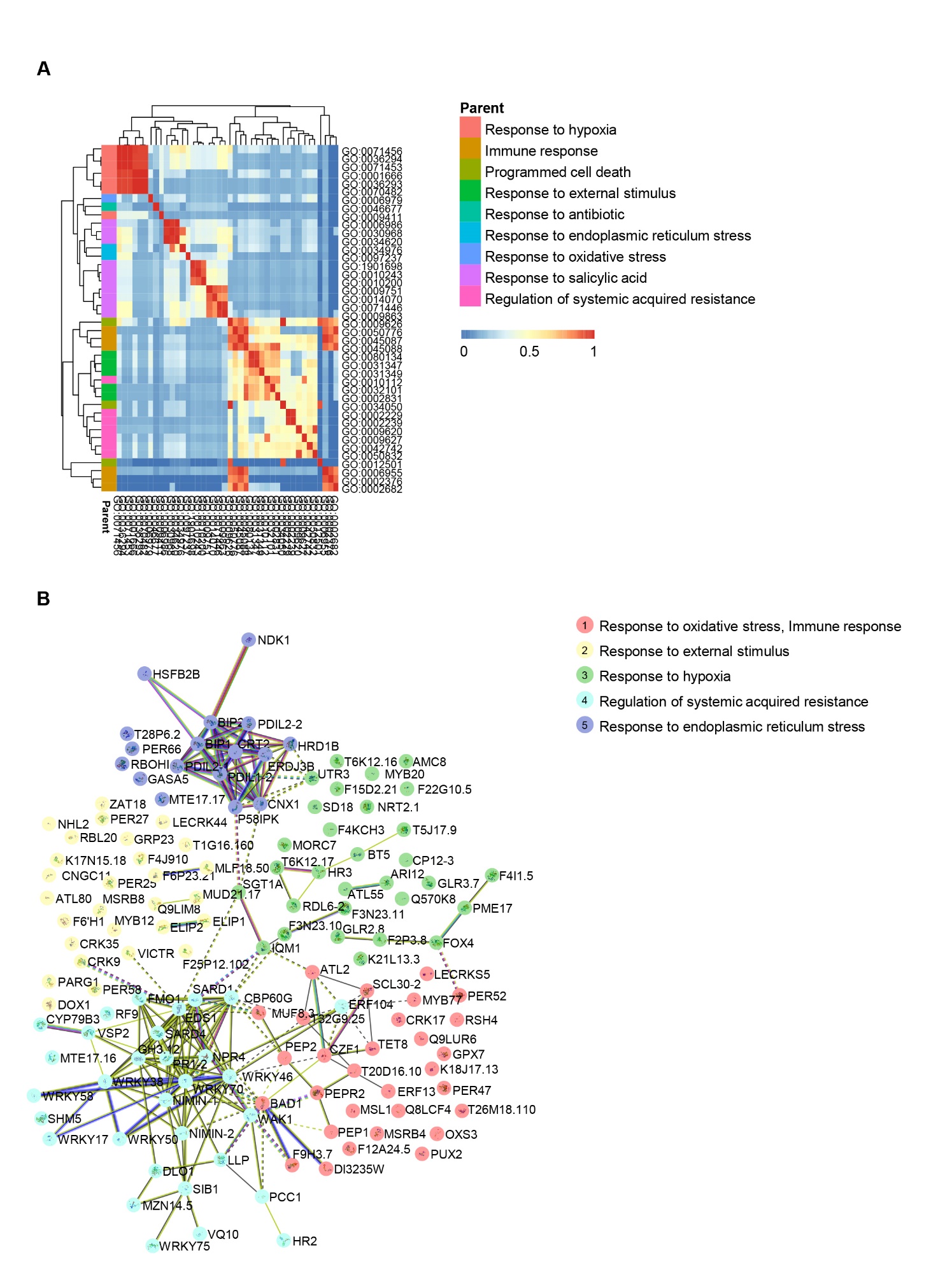
**

**Supplementary Figure 9.** Integrated analysis of enriched biological processes and protein-protein interactions among downregulated genes in the shoots of Al+POT1-treated plants. (A) Gene ontology (GO) analysis highlighting enriched biological processes. (B) Mapping of differentially down-regulated genes to the STRING database, revealing significant interactions (score >0.4). Nodes represent proteins, and connecting lines indicate specific protein-protein associations contributing to shared functions, not necessarily physical binding. Dotted lines connect distinct clusters. Line colors signify the type of interaction evidence: cornflower blue for known interactions from curated databases, purple for experimentally determined interactions, green for predictions based on gene neighborhood, red for predicted interactions from gene fusions, navy blue for predictions from gene co-occurrence, light green for predictions from text mining, olympic-blue for predictions from protein homology, and black for predictions based on co-expression. The network was further segmented into five specified clusters using kmeans clustering.

**
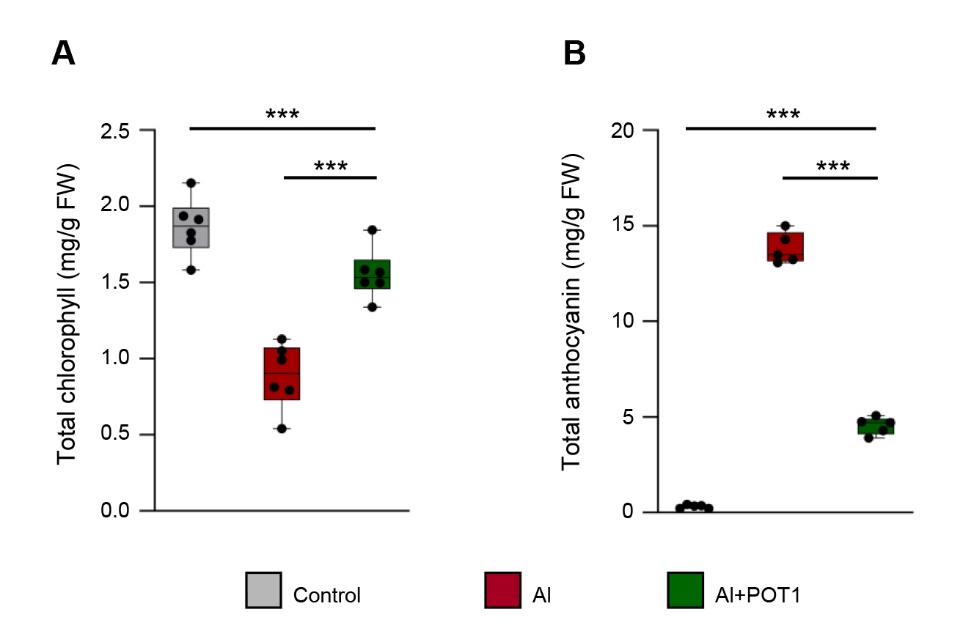
**

**Supplementary Figure 10.** POT1 alters chlorophyll and anthocyanin levels in *Arabidopsis*. Analysis of total chlorophyll content (A) and total anthocyanin content (B) of *Arabidopsis* seedlings subjected to different treatments: control (0mM AlCl_3_), Al stress (50mM AlCl_3_), or Al stress with POT1 inoculation (Al+POT1). Data were aggregated from 54 plants across three independent experiments, with 9 plants per data point. Boxplots depict upper and lower quartiles, while whiskers represent the range from the minimum to the lower quartile and from maximum to upper quartiles. Statistical significance of Al+POT1-treatment compared to control and Al-treatment was determined by Student’s *t*-test (*** *p* < 0.001). Detailed *p* values are provided in Supplementary Table 13.

**
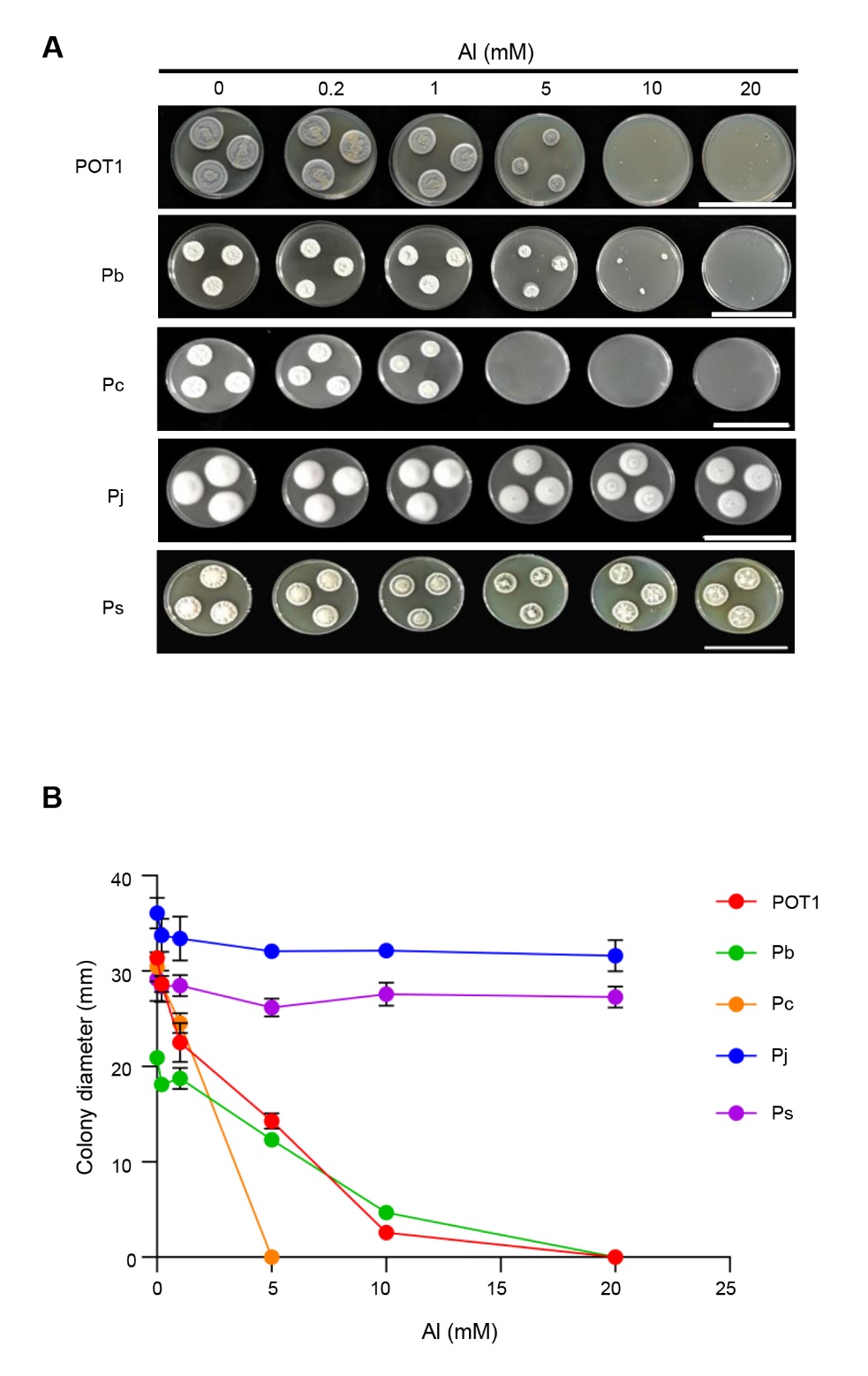
**

**Supplementary Figure 11.** Comparative assessment of Al tolerance between POT1 and other *Penicillium* species. Representative images (A) and measurements of colony diameter (B) of POT1 and various Al-tolerant *Penicillium* species on agar medium with five different Al concentrations (0.2, 1, 5, 10, and 20mM), relative to the control (0mM). The abbreviations used are Pb for *Penicillium bilaiae*, Pc for *Penicillium chrysogenum*, Pj for *Penicillium janthinellum,* Ps for *Penicillium simplicissimum.* Scale bar, 10cm.


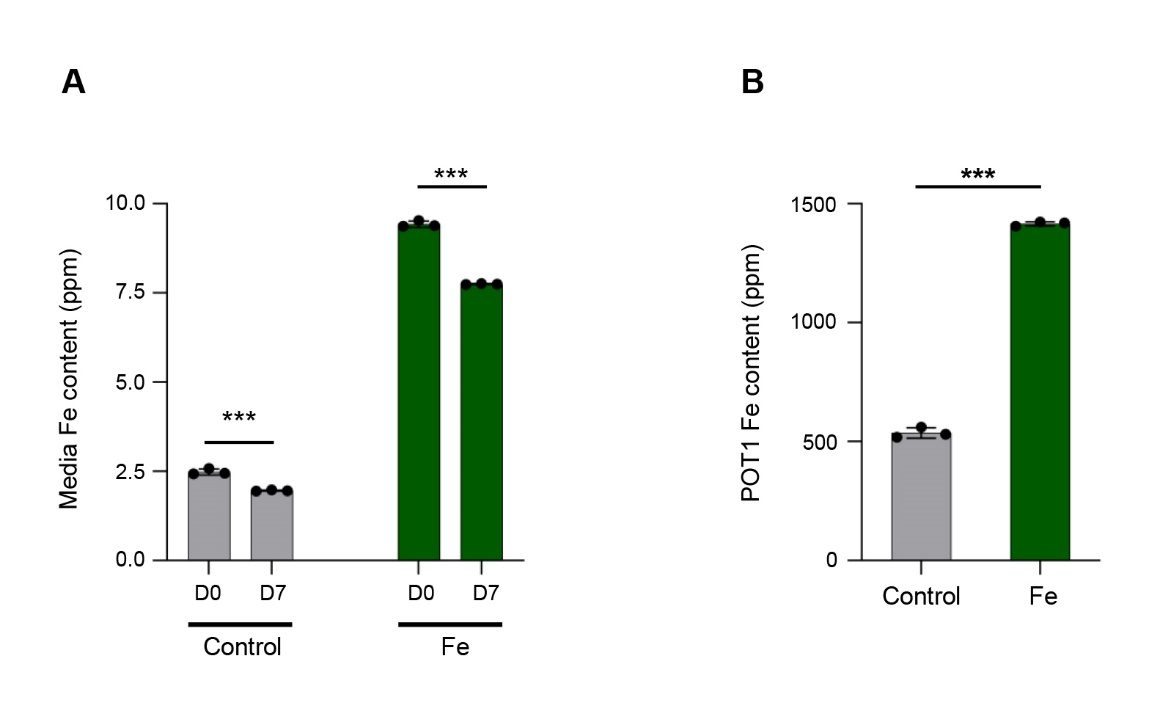


**Supplementary Figure 12.** POT1 internalizes iron from the media. (A) Comparison of Iron (Fe) content in spent media between control (0µM FeSO_4_) and Fe-treated (100µM FeSO_4_) media on day 0 (D0) and day 7 (D7). (B) Assessment of Fe content in POT1 mycelia cultured in control or Fe-treated media on D7. Columns represent mean ± SD of three independent experiments. Statistical significances were determined using Student’s *t*-test (*** *p* < 0.001). Detailed *p* values are provided in Supplementary Table 15.

**
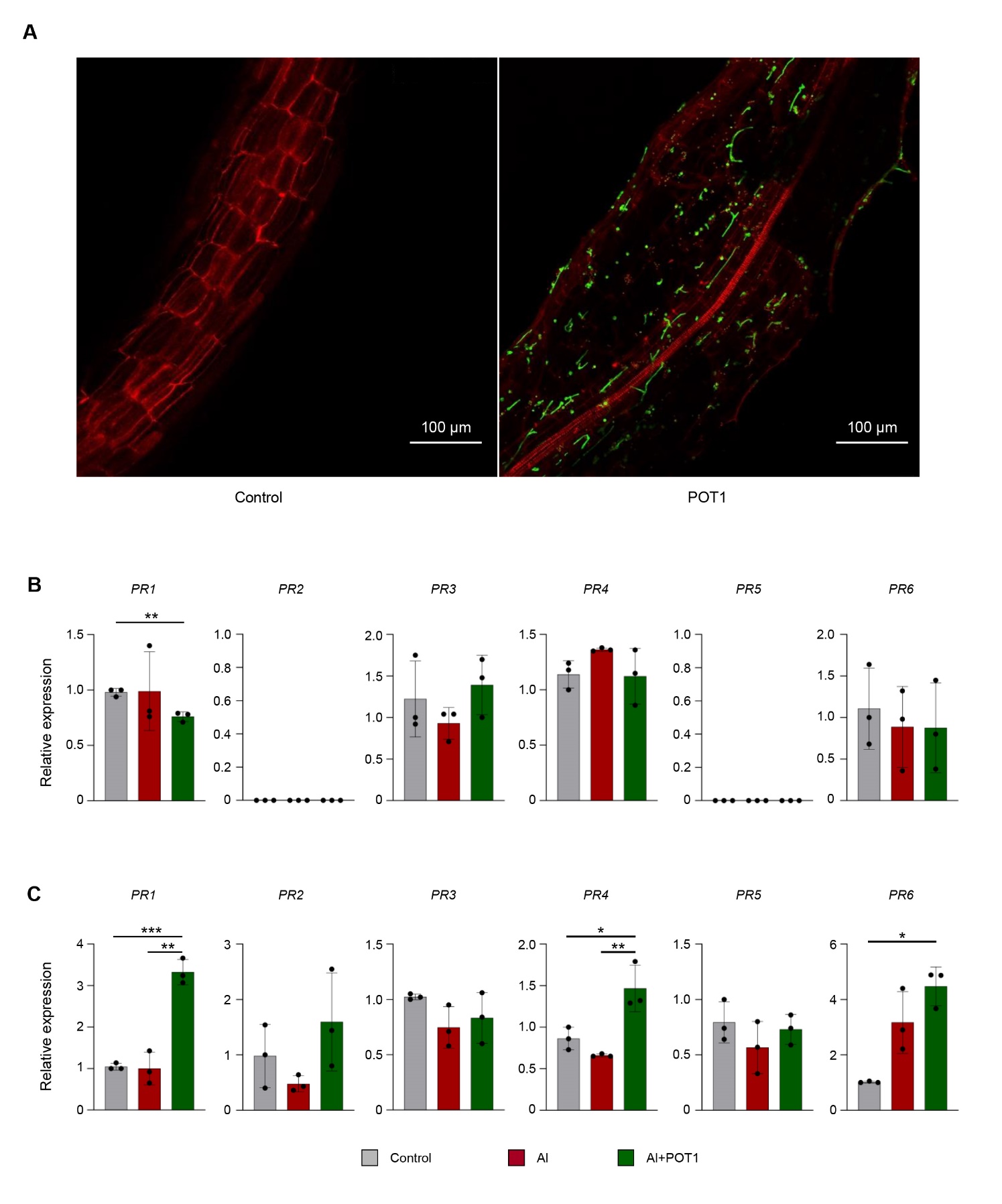
**

**Supplementary Figure 13.** POT1 establishes in root cells without inducing pathogenesis-related (*PR*) genes. (A) Seven days post-inoculation with POT1, roots samples were cleared and double-stained with propidium iodide (red) to visualize the cell wall and WGA-AF488 (green) to visualize fungal structures, imaged at 20x magnification. In control plants, only the cell wall (red) was observable, whereas in POT1-treated plants, both the cell wall (red) and intracellular hyphae (green) were detected. Scale bars, 100µm. (B and C) Relative expression levels of *PR* genes in the roots (B) and shoots (C) of Arabidopsis seedlings subjected to different treatments: control (0µM AlCl_3_), Al stress (100µM AlCl_3_), or Al stress with POT1 colonies (Al+POT1) at pH 4. Expression levels are normalized to the mean expression of *actin2*. Columns represent mean ± SD of three independent experiments. Statistical significance of Al+POT1-treatment compared to control and Al-treatment was determined using Student’s *t*-test (*** *p* < 0.001, ** *p* < 0.01, * *p* < 0.05). Detailed *p* values are provided in Supplementary Table 16.

**Supplementary Tables**

**Supplementary Table 1.** Primers used in this study

| Gene Locus | Gene Name | Primer Sequences | |
| --- | --- | --- | --- |
| AT2G14610 | *PR1* | F | 5′-TGATCCTCGTGGGAATTATGT-3' |
|  |  | R | 5′-TGCATGATCACATCATTACTTCAT-3' |
| AT3G57260 | *PR2* | F | 5′-AGCTTCCTTCTTCAACCACACAGC-3' |
|  |  | R | 5′-TGGCAAGGTATCGCCTAGCATC-3' |
| AT3G54420 | *PR3* | F | 5′-TCTAGCTTGAACGGTGGCTGTG-3' |
|  |  | R | 5′-CGTGCCATTAACGGTGCTTTGG-3' |
| AT3G04720 | *PR4* | F | 5′-TCCAAATCCAAGCCTCCGTTGC-3' |
|  |  | R | 5′-GCGGCAAGTGTTTAAGGGTGAAG-3' |
| AT1G75040 | *PR5* | F | 5′-AGCAATGCCGCTTGTGATGAAC-3' |
|  |  | R | 5′-ATCACCCACAGCACAGAGACAC-3' |
| AT2G38900 | *PR6* | F | 5′-ATACATGTCTAGCCGGCGGTTG-3' |
|  |  | R | 5′-TTGCTACCGCGTTAGGGTTTGG-3' |
| AT3G18780 | *actin2* | F | 5'-GTACAACCGGTATTGTGCTGGAT-3' |
|  |  | R | 5'-GCTTGGTGCAAGTGCTGTGATTTC-3' |

**Supplementary Table 2.** The *p* values for Figure 1

| Comparison | Day | Media Al content (C) | POT1 Al content (D) |
| --- | --- | --- | --- |
| Control and POT1 | Day 0 (0D) | < 0.0001 | NA |
| Control and POT1 | Day 7 (7D) | 0.0248 | < 0.0001 |

NA – not applicable

**Supplementary Table 3.** The *p* values for Figure 2

| Comparison | pH | Primary root length (B, G) | Root fresh weight (C, H) | Shoot fresh weight (D, I) | Leaf #2 Area (E, J) |
| --- | --- | --- | --- | --- | --- |
| Control and Al+POT1 | 4 | 0.0004 | 0.0002 | 0.0003 | 0.0002 |
|  | 5.6 | 0.0026 | 0.9543 | 0.1253 | 0.0840 |
| Al and Al+POT1 | 4 | < 0.0001 | 0.0005 | 0.0001 | < 0.0001 |
|  | 5.6 | 0.0021 | 0.1208 | 0.0002 | 0.0107 |

**Supplementary Table 4.** The *p* values for Supplementary Figure 2

| Comparison | Root Al content (A) | Shoot Al content (B) |
| --- | --- | --- |
| Control and Al+POT1 | 0.0018 | 0.3724 |
| Al and Al+POT1 | 0.0000 | 0.0385 |

**Supplementary Table 5.** The *p* values for Supplementary Figure 3

| pH | Comparison |  | S/R |
| --- | --- | --- | --- |
| 4 | Control and Al+POT1 |  | 0.0060 |
|  | Al and Al+POT1 |  | 0.0001 |
| 5.6 | Control and Al+POT1 |  | 0.1116 |
|  | Al and Al+POT1 |  | 0.0001 |

**Supplementary Table 6.** The *p* values for Figure 3

| Tissue | Comparison | N | P | K | Ca | Mg | B | Co | Cu |
| --- | --- | --- | --- | --- | --- | --- | --- | --- | --- |
| Root (A) | Control and Al+POT1 | 0.7527 | 0.0172 | 0.0314 | 0.3238 | 0.0071 | 0.0100 | 0.2242 | 0.0025 |
|  | Al and Al+POT1 | 0.1840 | 0.0110 | 0.6445 | 0.7740 | 0.0545 | 0.1549 | 0.5794 | 0.0740 |
| Shoot (B) | Control and Al+POT1 | 0.0314 | 0.0059 | 0.0043 | 0.0503 | 0.1624 | 0.0242 | 0.1087 | 0.2130 |
|  | Al and Al+POT1 | 0.0429 | 0.0006 | 0.4730 | 0.0974 | 0.0549 | 0.1392 | 0.7777 | 0.1736 |

| Tissue | Comparison | Fe | Mn | Mo | Na | S | Se | Zn |
| --- | --- | --- | --- | --- | --- | --- | --- | --- |
| Root (A) | Control and Al+POT1 | 0.0001 | 0.0066 | 0.0061 | 0.2400 | 0.3346 | 0.0030 | 0.0037 |
|  | Al and Al+POT1 | 0.0001 | 0.0031 | 0.0624 | 0.6046 | 0.5189 | 0.0463 | 0.7611 |
| Shoot (B) | Control and Al+POT1 | 0.0115 | 0.0685 | 0.6673 | 0.1204 | 0.6127 | 0.0193 | 0.0169 |
|  | Al and Al+POT1 | 0.1094 | 0.0650 | 0.0420 | 0.0312 | 0.1136 | 0.6094 | 0.0452 |

**Supplementary Table 7.** The *p* values for Supplementary Figure 4

| Tissue | Comparison | C | H | C/N |
| --- | --- | --- | --- | --- |
| Root (A) | Control and Al+POT1 | 0.8331 | 0.8566 | 0.5861 |
|  | Al and Al+POT1 | 0.0190 | 0.0665 | 0.8710 |
| Shoot (B) | Control and Al+POT1 | 0.0481 | 0.0915 | <0.0001 |
|  | Al and Al+POT1 | 0.0503 | 0.1517 | <0.0001 |

**Supplementary Table 8.** Overview of the RNA-Seq data

| Sample  Description | Sample ID | Raw reads | Raw bases (G) | Clean reads | Clean bases (G) | Error Rate (%) | Q20 (%) | Q30 (%) | GC(%) |
| --- | --- | --- | --- | --- | --- | --- | --- | --- | --- |
| Al-treated roots | Al-R1 | 93257728 | 13.99 | 91016854 | 13.65 | 0.03 | 97.56 | 92.92 | 43.62 |
|  | Al-R2 | 80207020 | 12.03 | 78873912 | 11.83 | 0.03 | 97.89 | 93.8 | 44.07 |
|  | Al-R3 | 87852160 | 13.18 | 86209336 | 12.93 | 0.03 | 97.37 | 92.55 | 43.74 |
| Al+POT1-treated roots | Al+POT1-R1 | 84528894 | 12.68 | 82497448 | 12.37 | 0.03 | 97.63 | 93.32 | 43.48 |
|  | Al+POT1-R2 | 93348324 | 14 | 91144518 | 13.67 | 0.03 | 97.83 | 93.74 | 44.52 |
|  | Al+POT1-R3 | 90399374 | 13.56 | 88525544 | 13.28 | 0.03 | 97.85 | 93.75 | 44.23 |
| Al-treated shoots | Al-S1 | 91452162 | 13.72 | 89814630 | 13.47 | 0.03 | 97.88 | 93.82 | 45.13 |
|  | Al-S2 | 86255174 | 12.94 | 85150174 | 12.77 | 0.03 | 97.39 | 92.55 | 45.35 |
|  | Al-S3 | 86372516 | 12.96 | 85196006 | 12.78 | 0.03 | 97.77 | 93.48 | 45.52 |
| Al+POT1-treated shoots | Al+POT1-S1 | 83452940 | 12.52 | 82221804 | 12.33 | 0.03 | 97.95 | 93.92 | 45.58 |
|  | Al+POT1-S2 | 84378982 | 12.66 | 82526654 | 12.38 | 0.03 | 97.79 | 93.54 | 45.36 |
|  | Al+POT1-S3 | 85117702 | 12.77 | 83430810 | 12.51 | 0.03 | 97.69 | 93.16 | 45.53 |

**Supplementary Table 9.** Overview of clean reads and mapped genes

| Sample | Sample_ID | Input reads | Mapped rate (%) | Unique mapping rate (%) | Multiple mapping rate (%) | Read1 mapping rate (%) | Read2 mapping rate (%) |
| --- | --- | --- | --- | --- | --- | --- | --- |
| Al-treated roots | Al-R1 | 91016854 | 95.68 | 93.67 | 2.01 | 46.78 | 46.88 |
|  | Al-R2 | 78873912 | 97.29 | 95.23 | 2.06 | 47.6 | 47.63 |
|  | Al-R3 | 86209336 | 97.92 | 95.71 | 2.21 | 47.9 | 47.81 |
| Al+POT1-treated roots | Al+POT1-R1 | 82497448 | 96.64 | 94.77 | 1.88 | 47.37 | 47.4 |
|  | Al+POT1-R2 | 91144518 | 97.11 | 94.87 | 2.24 | 47.42 | 47.45 |
|  | Al+POT1-R3 | 88525544 | 97.79 | 95.82 | 1.97 | 47.89 | 47.93 |
| Al-treated shoots | Al-S1 | 89814630 | 98.22 | 95.92 | 2.3 | 47.98 | 47.94 |
|  | Al-S2 | 85150174 | 98.3 | 96.14 | 2.16 | 48.19 | 47.95 |
|  | Al-S3 | 85196006 | 98.47 | 96.16 | 2.31 | 48.12 | 48.04 |
| Al+POT1-treated shoots | Al+POT1-S1 | 82221804 | 98.59 | 96.34 | 2.25 | 48.16 | 48.18 |
|  | Al+POT1-S2 | 82526654 | 98.4 | 96.06 | 2.34 | 48.06 | 48.01 |
|  | Al+POT1-S3 | 83430810 | 98.48 | 96.19 | 2.3 | 48.11 | 48.08 |

**Supplementary Table 10.** Expression levels of plant genes related to callose deposition in roots

| Gene ID | Gene Name | Gene Description | log_2_FC | *p* value |
| --- | --- | --- | --- | --- |
| AT5G36870 | *CALS4* | Callose synthase 4 | 0.260 | 0.0079 |
| AT1G05570 | *CALS1* | Callose synthase 1 | 0.190 | 0.0405 |
| AT3G14570 | *CALS8* | Callose synthase 8 | 0.149 | 0.0073 |
| AT4G04970 | *CALS11* | Callose synthase 11 | 0.128 | 0.0064 |
| AT3G59100 | *CALS6* | Callose synthase 6 | 0.118 | 0.0077 |
| AT4G03550 | *CALS12* | Callose synthase 12 | 0.118 | 0.0692 |
| AT2G36850 | *CALS10* | Callose synthase 10 | 0.102 | 0.0706 |
| AT3G14780 | *-* | Callose synthase | 0.060 | 0.0072 |
| AT5G13000 | *CALS3* | Callose synthase 3 | 0.036 | 0.0914 |
| AT2G31960 | *CALS2* | Callose synthase 2 | -0.004 | 0.0977 |
| AT3G58100 | *PDCB5* | Plasmodesmata callose-binding protein 5 | -0.115 | 0.0070 |
| AT1G18650 | *PDCB3* | Plasmodesmata callose-binding protein 3 | -0.150 | 0.0052 |
| AT1G06490 | *CALS7* | Callose synthase 7 | -0.243 | 0.0042 |
| AT1G69295 | *PDCB4* | Plasmodesmata callose-binding protein 4 | -0.294 | 0.0118 |
| AT2G13680 | *CALS5* | Callose synthase 5 | -0.450 | 0.0068 |
| AT3G57270 | *BG1* | Glucan endo-1,3-beta-glucosidase | 2.185 | 0.0052 |
| AT4G16260 | *-* | Glucan endo-1,3-beta-glucosidase | 0.983 | 0.0020 |
| AT5G20330 | *BG4* | Glucan endo-1,3-beta-glucosidase | 0.863 | 0.0072 |
| AT2G27500 | *-* | Glucan endo-1,3-beta-glucosidase 14 | 0.206 | 0.0546 |
| AT1G11820 | *-* | Glucan endo-1,3-beta-glucosidase 1 | 0.175 | 0.0562 |
| AT5G58480 | *-* | Glucan endo-1,3-beta-glucosidase 9 | 0.150 | 0.0046 |
| AT5G58090 | *-* | Glucan endo-1,3-beta-glucosidase 6 | 0.069 | 0.0075 |
| AT4G31140 | *-* | Glucan endo-1,3-beta-glucosidase 5 | 0.044 | 0.0084 |
| AT5G42100 | *ATBG_PPAP* | Glucan endo-1,3-beta-glucosidase 10 | 0.041 | 0.0856 |
| AT1G32860 | *-* | Glucan endo-1,3-beta-glucosidase 11 | -0.024 | 0.0939 |
| AT2G01630 | *-* | Glucan endo-1,3-beta-glucosidase 3 | -0.083 | 0.0062 |
| AT3G13560 | *-* | Glucan endo-1,3-beta-glucosidase 4 | -0.130 | 0.0049 |
| AT1G64760 | *-* | Glucan endo-1,3-beta-glucosidase 8 | -0.204 | 0.0565 |
| AT5G56590 | *-* | Glucan endo-1,3-beta-glucosidase 13 | -0.215 | 0.0010 |
| AT4G34480 | *-* | Glucan endo-1,3-beta-glucosidase 7 | -0.294 | 0.0437 |
| AT1G61065 | *-* | 1,3-Beta-glucan synthase | -0.324 | 0.0353 |
| AT1G66250 | *-* | Glucan endo-1,3-beta-glucosidase 2 | -0.359 | 0.0004 |
| AT5G08000 | *E13L3* | Glucan endo-1,3-beta-glucosidase-like protein 3 | -0.364 | 0.0054 |

**Supplementary Table 11.** Expression levels of plant genes related to nutrient transportation

| Gene ID | Gene Name | Gene Description | log_2_FC | *p* value |
| --- | --- | --- | --- | --- |
| **Roots** | | | | |
| AT5G52710 | *-* | Copper transporter | 2.412 | 7.68E-05 |
| AT5G43360 | *PHT1-3* | Inorganic phosphate transporter 1-3 | 1.961 | 1.15E-05 |
| AT5G52720 | *-* | Copper transporter | 1.561 | 0.0412 |
| AT4G00910 | *-* | Aluminium activated malate transporter | 1.355 | 0.0430 |
| AT5G43370 | *PHT1-2* | Inorganic phosphate transporter 1-2 | 1.310 | 0.0111 |
| AT1G28010 | *ABCB14* | ABC transporter B family member 14 | 1.308 | 0.0080 |
| AT4G10310 | *HKT1* | Sodium transporter | 1.290 | 0.0300 |
| AT1G79360 | *OCT2* | Organic cation/carnitine transporter 2 | 1.228 | 0.0019 |
| AT2G25680 | *MOT1* | Molybdate transporter 1 | 1.183 | 0.0121 |
| AT1G68740 | *PHO1-H1* | Phosphate transporter PHO1 homolog 1 | 1.117 | 0.0250 |
| AT1G76430 | *PHT1-9* | Inorganic phosphate transporter 1-9 | 1.114 | 0.0126 |
| AT1G11540 | *-* | Sulfite exporter TauE/SafE family protein | 0.967 | 0.0014 |
| AT3G63380 | *ACA12* | Calcium-transporting ATPase | 0.937 | 0.0115 |
| AT1G09930 | *OPT2* | Oligopeptide transporter 2 | 0.838 | 0.0048 |
| AT2G18480 | *PLT3* | Probable polyol transporter 3 | 0.817 | 0.0296 |
| AT5G43350 | *PHT1-1* | Inorganic phosphate transporter 1-1 | 0.727 | 0.0029 |
| AT3G59140 | *ABCC10* | ABC transporter C family member 10 | 0.713 | 0.0097 |
| AT4G21903 | *-* | MATE efflux family protein | 0.671 | 0.0322 |
| AT3G14770 | *SWEET2* | Bidirectional sugar transporter | 0.635 | 0.0177 |
| AT1G80830 | *NRAMP1* | Metal transporter | 0.631 | 0.0001 |
| AT1G61740 | *-* | Sulfite exporter TauE/SafE family protein 2 | 0.557 | 0.0014 |
| AT3G47780 | *ABCA7* | ABC transporter A family member 7 | 0.514 | 0.0018 |
| AT1G80510 | *AVT6E* | Amino acid transporter | 0.503 | 0.0206 |
| AT1G02520 | *ABCB11* | ABC transporter B family member 11 | 0.478 | 0.0277 |
| AT3G57330 | *ACA11* | Calcium-transporting ATPase 11, plasma membrane-type | 0.430 | 0.0329 |
| AT3G23430 | *PHO1* | Phosphate transporter PHO1 | 0.430 | 0.0030 |
| AT3G26590 | *DTX29* | MATE efflux family protein | 0.397 | 0.0361 |
| AT5G65380 | *DTX27* | MATE efflux family protein | 0.389 | 0.0291 |
| AT1G75220 | *-* | Sugar transporter ERD6-like 6 | 0.354 | 0.0413 |
| AT5G04160 | *UUAT1* | UDP-Uronic acid transporter1 | -0.399 | 0.0327 |
| AT5G13550 | *SULTR4;1* | Sulfate transporter 4.1 | -0.492 | 0.0051 |
| AT3G12520 | *SULTR4;2* | Sulfate transporter 4.2 | -0.592 | 0.0152 |
| AT2G38170 | *CAX1* | Vacuolar cation/proton exchanger | -0.620 | 0.0112 |
| AT5G10180 | *SULTR2;1* | Sulfate transporter 2.1 | -0.726 | 0.0094 |
| AT1G80760 | *NIP6-1* | Aquaporin NIP6-1 | -0.733 | 0.0258 |
| AT3G56290 | *-* | Potassium transporter | -0.744 | 0.0092 |
| AT2G04080 | *DTX2* | MATE efflux family protein | -0.848 | 0.0037 |
| AT1G23000 | *-* | Heavy metal transport/detoxification superfamily protein | -0.939 | 0.0109 |
| AT3G51895 | *SULTR3;1* | Sulfate transporter 3.1 | -0.989 | 0.0333 |
| AT5G50800 | *SWEET13* | Bidirectional sugar transporter | -1.108 | 0.0117 |
| AT1G71140 | *DTX14* | MATE efflux family protein | -1.169 | 0.0420 |
| AT5G55930 | *OPT1* | Oligopeptide transporter 1 | -1.388 | 0.0204 |
| AT4G01830 | *ABCB5* | ABC transporter B family member 5 | -1.397 | 0.0011 |
| AT5G65990 | *AVT3A* | Amino acid transporter | -1.402 | 0.0000 |
| AT3G25620 | *ABCG21* | ABC transporter G family member 21 | -1.506 | 0.0267 |
| AT4G25010 | *SWEET14* | Bidirectional sugar transporter | -1.535 | 0.0036 |
| AT2G33280 | *-* | Folate-biopterin transporter 9 | -1.542 | 0.0132 |
| AT1G79900 | *BAC2* | Mitochondrial arginine transporter | -1.650 | 0.0006 |
| AT5G60770 | *NRT2.4* | High affinity nitrate transporter 2.4 | -1.735 | 9.74E-07 |
| AT5G06530 | *ABCG22* | ABC transporter G family member 22 | -1.742 | 3.88E-07 |
| AT4G26590 | *OPT5* | Oligopeptide transporter 5 | -2.258 | 0.0387 |
| AT1G73700 | *DTX17* | MATE efflux family protein | -3.130 | 0.0010 |
| **Shoots** | | | | |
| AT1G69480 | *PHO1-H10* | Phosphate transporter PHO1 homolog 10 | 0.859 | 0.0028 |
| AT5G18840 | *-* | Sugar transporter ERD6-like 16 | 0.834 | 0.0257 |
| AT3G21090 | *ABCG15* | ABC transporter G family member 15 | 0.797 | 0.0206 |
| AT5G10820 | *-* | Folate-biopterin transporter 6 | 0.545 | 0.0122 |
| AT1G51090 | *-* | Heavy metal transport/detoxification superfamily protein | 0.521 | 0.0060 |
| AT5G15410 | *CNGC2* | Cyclic nucleotide-gated ion channel 2 | 0.514 | 0.0009 |
| AT2G39130 | *AVT1C* | Amino acid transporter | 0.464 | 0.0041 |
| AT2G36630 | *-* | Sulfite exporter TauE/SafE family protein 4 | 0.449 | 0.0330 |
| AT1G08230 | *GAT1* | GABA transporter 1 | 0.441 | 0.0056 |
| AT3G47420 | *ATPS3* | Glycerol-3-phosphate transporter 1 | 0.416 | 0.0233 |
| AT2G36380 | *ABCG34* | ABC transporter G family member 34 | 0.313 | 0.0324 |
| AT2G41560 | *ACA4* | Calcium-transporting ATPase 4, plasma membrane-type | 0.308 | 0.0371 |
| AT1G79160 | *-* | Filamentous hemagglutinin transporter | 0.304 | 0.0210 |
| AT5G24030 | *SLAH3* | S-type anion channel | 0.273 | 0.0369 |
| AT5G21930 | *PAA2* | Copper-transporting ATPase, chloroplastic | 0.258 | 0.0270 |
| AT5G27150 | *NHX1* | Sodium/hydrogen exchanger | -0.315 | 0.0308 |
| AT5G04160 | *UUAT1* | UDP-Uronic acid transporter 1 | -0.316 | 0.0357 |
| AT3G56290 | *-* | Potassium transporter | -0.318 | 0.0362 |
| AT1G09930 | *OPT2* | Oligopeptide transporter 2 | -0.414 | 0.0197 |
| AT5G23660 | *SWEET12* | Bidirectional sugar transporter | -0.454 | 0.0210 |
| AT3G45650 | *NAXT1* | Nitrate excretion transporter 1 | -0.582 | 0.0168 |
| AT3G13100 | *ABCC7* | ABC transporter C family member 7 | -0.593 | 0.0349 |
| AT1G14360 | *UTR3* | UDP-galactose/UDP-glucose transporter 3 | -0.678 | 0.0252 |
| AT3G21080 | *-* | ABC transporter-like protein | -0.846 | 0.0263 |
| AT5G60800 | *-* | Heavy metal transport/detoxification superfamily protein | -0.880 | 0.0015 |
| AT1G02470 | *-* | Polyketide cyclase/dehydrase and lipid transport superfamily protein | -1.127 | 0.0279 |
| AT1G08090 | *NRT2.1* | High-affinity nitrate transporter 2.1 | -1.487 | 0.0034 |
| AT1G73220 | *OCT1* | Organic cation/carnitine transporter 1 | -2.061 | 0.0173 |
| AT1G30473 | *-* | Heavy metal transport/detoxification superfamily protein | -3.742 | 0.0390 |

**Supplementary Table 12.** The *p* values for Figure 4

| Comparison | Shoot fresh weight (C) | Al content (D) | Shoot fresh weight (G) | Al content (H) |
| --- | --- | --- | --- | --- |
| Control and Al+POT1 | 0.0263 | 0.0015 | < 0.0001 | < 0.0001 |
| Al and Al+POT1 | < 0.0001 | 0.0005 | < 0.0001 | 0.0133 |

**Supplementary Table 13.** The *p* values for Supplementary Figure 10

| Comparison | Total chlorophyll (A) | Total anthocyanin (B) |
| --- | --- | --- |
| Control and Al+POT1 | 0.0001 | < 0.0001 |
| Al and Al+POT1 | < 0.0001 | < 0.0001 |

**Supplementary Table 14.** The *p* values for Figure 5

| Comparison | Primary root length (B) | Root fresh weight (C) | Shoot fresh weight (D) | Total anthocyanin (F) | Total chlorophyll (G) |
| --- | --- | --- | --- | --- | --- |
| Control and POT1 | < 0.0001 | 0.0003 | <0.0001 | < 0.0001 | < 0.0001 |
| Pb and POT1 | 0.7090 | 0.6794 | 0.0352 | 0.0190 | < 0.0001 |
| Pc and POT1 | 0.2800 | 0.0104 | 0.0065 | 0.0098 | 0.0014 |
| Pj and POT1 | 0.3561 | 0.0438 | 0.6689 | 0.0004 | < 0.0001 |
| Ps and POT1 | 0.0496 | 0.0039 | 0.0005 | 0.0009 | < 0.0001 |

**Supplementary Table 15.** The *p* values for Supplementary Figure 12

| Comparison | Day | Media Fe content (A) | POT1 Fe content (B) |
| --- | --- | --- | --- |
| Control and Fe media | Day 0 | < 0.0001 | NA |
| Control and Fe media | Day 7 | < 0.0001 | < 0.0001 |
| NA - not applicable |  |  |  |

**Supplementary Table 16.** The *p* values for Supplementary Figure 13

| Tissue | Comparison | *PR1* | *PR2* | *PR3* | *PR4* | *PR5* | *PR6* |
| --- | --- | --- | --- | --- | --- | --- | --- |
| Root (A) | Control and Al+POT1 | 0.0024 | NA | 0.6391 | 0.9230 | NA | 0.6132 |
|  | Al and Al+POT1 | 0.3289 | NA | 0.1189 | 0.1737 | NA | 0.9821 |
| Shoot (B) | Control and Al+POT1 | 0.0002 | 0.3679 | 0.2281 | 0.0284 | 0.6581 | 0.0010 |
|  | Al and Al+POT1 | 0.0013 | 0.0967 | 0.6398 | 0.0076 | 0.3557 | 0.1615 |

NA – not applicable
